# Thermodynamic Parameters of Berberine with Kolliphor Mixtures Adsorption and Micellization

**DOI:** 10.3390/molecules28073115

**Published:** 2023-03-30

**Authors:** Magdalena Szaniawska, Katarzyna Szymczyk, Anna Zdziennicka, Bronisław Jańczuk

**Affiliations:** Department of Interfacial Phenomena, Institute of Chemical Sciences, Faculty of Chemistry, Maria Curie-Skłodowska University in Lublin, Maria Curie-Skłodowska Sq. 3, 20-031 Lublin, Poland

**Keywords:** berberine, Kolliphor^®^ ELP, Kolliphor^®^ RH 40, adsorption, micellization, thermodynamic parameters of adsorption and micellization

## Abstract

The poor solubility of berberine (Ber) in water limits its practical use. Its solubility can be increased, among other ways, by the addition of surfactants. Of the surfactants, Kolliphor^®^ ELP (ELP) and Kolliphor^®^ RH 40 (RH40) can be very useful in this respect. The increase of Ber’s solubility in water in the presence of ELP and RH40 should be reflected in the composition of the surface layers at the water-air interface and the micelles. The determined composition is reflected in the Gibbs energy of interactions of berberine with ELP and RH40 through the water phase and the standard Gibbs free energy, enthalpy, and entropy of adsorption and micellization. These energies were determined from the equations proposed by us, based on the Gibbs surface excess concentration of the Ber mixture with ELP and RH40, the activity of these compounds in the surface layer at the water-air interface and in the micelles obtained by the Hua and Rosen method, and the contributions of Ber, ELP, and RH40 to the reduction in the water surface tension. For this determination, the measurements of the surface tension of the aqueous solution of the Ber mixture with ELP or RH40 and that of the Ber mixture with these two surfactants, as well as the density and conductivity were performed. Moreover, the fluorescence emission spectra for the Ber + surfactant mixtures were recorded.

## 1. Introduction

**Figure 1:**
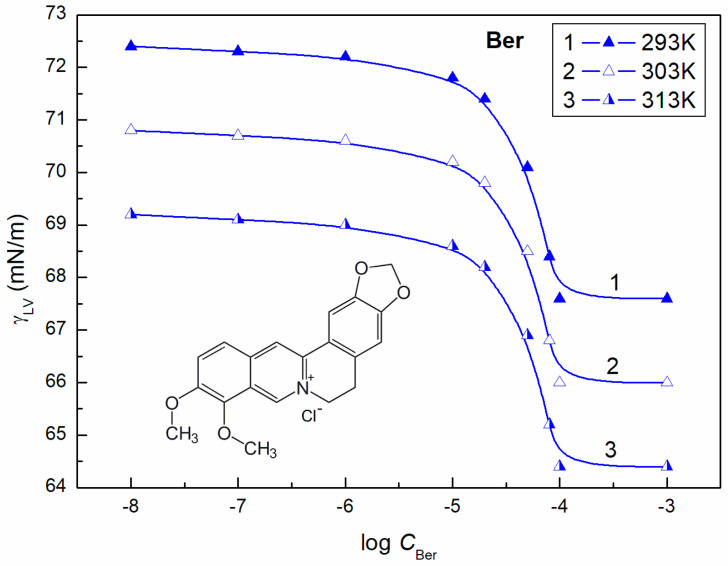
A plot of the surface tension ($\gamma_{LV}$) of berberine (Ber) aqueous solutions vs. the logarithm of Ber concentration (log $C_{\mathrm{Ber}}$) at a constant temperature equal to 293 K (curve 1), 303 K (curve 2), and 313 K (curve 3).

Berberine (Ber), which occurs in the roots, rhizomes, stems and bark of medicinal plants is widely used in medicine [1,2,3,4,5,6,7,8,9,10]. Its molecule consists of condensed hydrocarbon rings, a ring containing oxygen atoms, and a ring with a quaternary nitrogen atom, as well as the –CH_3_O groups (Figure 1). The predominance of hydrophobic groups over hydrophilic ones in the berberine molecule causes its poor solubility in water [11,12], which, among others, is the reason for the limited use of berberine in practice. The solubility of Ber in water can be increased, among other ways, by mixing it with suitable surfactants. Such surfactants include Kolliphor^®^ ELP (ELP) and Kolliphor^®^ RH 40 (RH40). These surfactants, which are well soluble in water, are nontoxic, allowing them to be used, for example, in cosmetics and pharmaceutical products [13,14,15,16]. The good solubility of ELP and RH40 results from the presence of many oxyethylene groups, as well as –C=O and =C–O groups in their molecules. However, in the ELP and RH40 molecules, hydrocarbon chains containing one –OH group are also present. This group decreases the hydrophobic properties of these chains to some extent.

The type and amount of the chemical groups present in the molecule determine the values of the compound surface tension and its components, as well as the number of the water molecules that can be contacted with this compound in aqueous media. According to van Oss and Constanzo [17], the surface tension of surfactants depends on their molecules’ orientation toward the air phase. If they are oriented toward this phase by the hydrophobic parts of their molecules, then the surfactant surface tension is treated as its tail surface tension. Otherwise, when the surfactant molecules are oriented by their hydrophilic part toward the air phase, then the surface tension is called the head surface tension. In turn, van Oss et al. divided the surface tension of all substances into the Lifshitz-van der Waals (LW) and acid-base (AB) components. The AB component associated with hydrogen bond formation is a function of electron-acceptor and electron-donor parameters of the surface tension [17,18,19,20]. For the condensed phases, van Oss et al. proved that the LW component results from the dispersion intermolecular interactions because the contributions of dipole-dipole and induced dipole-dipole interactions to this component are smaller than 2% [18,19,20].

Knowledge of the values of the components and parameters of the surfactants’ surface tension allows for the determination of the Gibbs free energy of interactions of the surfactants through the water phase. Additionally, knowing the contactable area of the hydrophobic and hydrophilic parts of the surfactants’ molecules, it is possible to predict their tendency to adsorb and aggregate [21,22]. In the other words, based on the components and parameters of the surfactants’ tail and head surface tension, as well as their contactable area, it is possible to predict the standard Gibbs free energy of adsorption and micellization. This process is possible for single surfactants. In the case of surfactant mixtures or their mixture with additives, it is more complicated. Moreover, such considerations are rarely found in the literature. In the case of such mixtures, the standard Gibbs energy of adsorption at the water-air interface and aggregation depends on the activity of all mixture components in the surface layer and in the micelles, which has not often been considered by investigators [23,24]. In fact, the activity depends on the composition and activity coefficient.

The knowledge of the interactions between Ber and ELP, as well as RH40 molecules in aqueous media associated with the standard Gibbs free energy of adsorption and micellization, should enable understanding of the increase in Ber solubility in water, which is important from the practical point of view. As it is difficult to find such studies, the aim of our paper was to determine the relationship among the intermolecular interactions of the Ber, ELP, and RH40 through the water phase and the relationship between the composition of the mixed surface layer and mixed micelles, as well as the standard Gibbs free energy of adsorption and micellization. To solve this problem, measurements of the surface tension, conductivity, and density at temperatures of 293, 303 and 313 K of the following solutions were performed: ELP + Ber (M1), RH40 + Ber (M2), and ELP + RH40 + Ber (M3). In each solution, the concentration of Ber was constant and equal to 1 × 10^−4^ mol/dm^3^, and the concentration of surfactants was in the range of 1 × 10^−6^–1 × 10^−2^ mol/dm^3^. In the M3 mixture, the mole fraction of ELP in the bulk phase was equal to 0.8. Moreover, the fluorescence emission spectra for the Ber + surfactant mixtures were recorded. Additionally, for the determination of the components and parameters of berberine’s surface tension, the contact angles of water, diiodomethane, and formamide on pressed Ber tablets were measured.

## 2. Theory

The amount of the surfactants adsorbed at the water-air (W-A) interface as well as the standard Gibbs free energy of adsorption and micellization, is directly associated with the chemical potential of the surfactants in the surface layer and/or micelles, as well as in the bulk phase. It should be remembered that, at a constant temperature (T) and pressure (p), the chemical potential of i-th solution component ($\mu_{i}$) is equal to the contribution of the Gibbs free energy ($G_{i}$) of this component. The adsorption and micellization processes of a single surfactant are related to the standard Gibbs free energy of these processes. In turn, the standard Gibbs free energy of adsorption ($\Delta G_{ads}^{0}$) and micellization ($\Delta G_{mic}^{0}$) is equal to the difference between the standard potential, which is symmetrically ($\mu_{i}^{0}$) and asymmetrically ($\mu_{i}^{*}$) defined. In the case of solutions of multicomponent mixture, the changes of the Gibbs free energy of the solution during the adsorption and micellization processes can be also related to the Gibbs free energy of surfactants mixing in the monolayer at the W-A interface and in the micelles.

In the case of aqueous solutions, the amount of surfactant mixture adsorbed at the W-A interface is connected to the changes in the solution surface tension ($\gamma_{LV}$), which is expressed by the Gibbs-Duhem equation [25,26]:

(1) $$Ad\gamma_{LV}+\sum_{i=j}^{i=1}n_{i}d\mu_{i}=0,$$

where $n_{i}$ is the number of moles of the *i*-th component, *A* is the area of the interface, and j is the number of the components. 

Knowing that, in the equilibrium state, the chemical potential of i-th component in the surface layer is equal to that in the bulk phase, assuming that the activity of j≠i components is constant and that the Gibbs surface excess concentration of i-th component, $\Gamma_{i}$, is equal to $\frac{n_{i}}{A}$, the following can be written:

(2) $$\Gamma_{i}=-\frac{a_{i}}{mRT}{\left(\frac{\partial \gamma_{LV}}{\partial a_{i}}\right)}_{a_{j\neq i},T}=-\frac{1}{mRT}{\left(\frac{\partial \gamma_{LV}}{\partial \ln a_{i}}\right)}_{a_{j\neq i},T},$$

where the value of *m* depends on the type of surfactant; for example, for the ionic surfactant type 1:1 electrolyte is equal to 2, $a_{i}$ is the activity, and *R* is the gas constant. 

If the concentration of surfactants is small, then according to the asymmetrical definition of the chemical potential, its activity coefficient ($f_{i}$) is close to unity, and $a_{i}≅x_{i}$ ($x_{i}$ is the molar fraction of i-th component in the bulk phase). In such cases, $x_{i}≅C_{i}/\omega$, where $C_{i}$ is the mole concentration of the i-th component in the bulk phase, and *ω* is the number of the water moles in 1 dm^3^ of solution. Taking these assumptions into account, Equation (2) can be written as:

(3) $$\Gamma_{i}=-\frac{C_{i}}{mRT}{\left(\frac{\partial \gamma_{LV}}{\partial C_{i}}\right)}_{C_{j\neq i},T}=-\frac{1}{2.303mRT}{\left(\frac{\partial \gamma_{LV}}{\partial \log C_{i}}\right)}_{C_{j\neq i},T},$$

In the case of the constant composition of the multicomponent mixtures of substrates in the bulk phase, it was proved that the sum of the mixture’s Gibbs surface excess concentration at the W-A interface ($\Gamma_{sum}$) can be determined from the following equation:

(4) $$\Gamma_{\mathrm{sum}}=-\frac{C_{sum}}{mRT}{\left(\frac{\partial \gamma_{LV}}{\partial C_{sum}}\right)}_{T}=-\frac{1}{2.303mRT}{\left(\frac{\partial \gamma_{LV}}{\partial \log C_{sum}}\right)}_{T},$$

where $C_{sum}$ is the sum of the mole concentration of all components of substrate in the solution.

Unfortunately, in the case of the mixtures in which the concentrations of all components except one in the bulk phase are constant, the Gibbs surface excess concentration at the W-A interface of the components at a constant concentration in the bulk phase changes as a function of component at variable concentration. In such cases, it is possible to determine the Gibbs surface concentration using the Frumkin equation, which for i-th component of the mixture has this form:

(5) $$\pi_{i}=-RT\Gamma_{i}^{max}\ln\left(1-\frac{\Gamma_{i}}{\Gamma_{i}^{max}}\right),$$

where $\pi_{i}$ is the contribution of i-th component to the reduction in water surface tension ($\gamma_{w}$), $\Gamma_{i}$ is the concentration of i-th component of the mixture at the W-A interface, and $\Gamma_{i}^{max}$ is the maximal concentration of i-th mixture component at the W-A interface.

It is proved that the fraction of area occupied by the i-th component in the mixture at the W-A interface ($x_{i}^{S}$) is equal to $\frac{\pi_{i}^{ind}}{\sum_{i=j}^{i=1}\pi_{i}^{ind}}$, where $\pi_{i}^{ind}$ is the film pressure at the W-A interface of the individual component of the mixture in the absence of others. Hence $\pi_{i}$ can be determined from the following expression [21]:

(6) $$\pi_{i}=x_{i}^{S}\pi =x_{i}^{S}\left(\gamma_{w}-\gamma_{LV}\right),$$

The studies of the composition of binary and ternary mixtures of surfactants have shown that the $x_{i}^{S}$ values determined based on the contributions of particular mixture components to the reduction in water surface tension are comparable to those determined from the Hua and Rosen concept [27]. The Hua and Rosen concept, based on the non-ideal solution theory, allows for determining the composition of the binary mixture of the surfactants in the interface region [27]. However, our studies showed that it is possible to apply the Hua and Rosen concept successfully to determine the composition of the mixed monolayer of the ternary surfactants’ mixture [21]. For the i-th, k-th, and l-th components of the ternary mixture, the Rosen and Hua equation can be written in this form:

(7) $$\frac{{\left(x_{ik}^{S}\right)}^{2}\ln\left(\alpha_{ik}C_{ikl}^{0}/x_{ik}^{S}C_{ik}^{0}\right)}{{\left(1-x_{ik}^{S}\right)}^{2}\ln\left[\left(1-\alpha_{ik}\right)C_{ikl}^{0}/\left(1-x_{ik}^{S}\right)C_{l}^{0}\right]}=1,$$

and

(8) $$\frac{{\left(x_{kl}^{S}\right)}^{2}\ln\left(\alpha_{kl}C_{ikl}^{0}/x_{kl}^{S}C_{kl}^{0}\right)}{{\left(1-x_{kl}^{S}\right)}^{2}\ln\left[\left(1-\alpha_{kl}\right)C_{ikl}^{0}/\left(1-x_{kl}^{S}\right)C_{i}^{0}\right]}=1,$$

and

(9) $$\frac{{\left(x_{il}^{S}\right)}^{2}\ln\left(\alpha_{il}C_{ikl}^{0}/x_{il}^{S}C_{il}^{0}\right)}{{\left(1-x_{il}^{S}\right)}^{2}\ln\left[\left(1-\alpha_{il}\right)C_{ikl}^{0}/\left(1-x_{il}^{S}\right)C_{k}^{0}\right]}=1,$$

where α is the mole fraction of a given component in the surfactant mixture in the bulk phase of the solution, $C^{0}$ is the concentration of the individual component of the mixture or the sum of components’ concentrations at which the water surface tension is reduced to the same value, and $\alpha_{ik}=\alpha_{i}+\alpha_{k}$, $\alpha_{il\;}=\alpha_{i}+\alpha_{l}$, $\alpha_{kl}=\alpha_{k}+\alpha_{l}$, $x_{ik}^{S}=x_{i}^{S}+x_{k}^{S}$, $x_{il}^{S}=x_{i}^{S}+x_{l}^{S}$, $x_{kl}^{S}=x_{l}^{S}+x_{k}^{S}$, $C_{ikl}^{0}=C_{i}^{0}+C_{k}^{0}\;$+$\;C_{l}^{0}$, $C_{ik}^{0}=C_{i}^{0}+C_{k}^{0}$, $C_{il}^{0}=C_{i}^{0}+C_{l}^{0}$ and $C_{kl}^{0}=C_{l}^{0}+C_{k}^{0}$.

The composition of the mixed micelles of binary mixtures of the surfactants in the aqueous media can be determined using the Rubingh equation [28,29]. This equation can be successfully used for the ternary surfactant mixture in a modified way [21]. For the ternary mixtures, it can be written as:

(10) $$\frac{{\left(x_{ik}^{M}\right)}^{2}\ln\left(\alpha_{ik}C_{ikl}^{M}/x_{ik}^{M}C_{ik}^{M}\right)}{{\left(1-x_{ik}^{M}\right)}^{2}\ln\left[\left(1-\alpha_{ik}\right)C_{ikl}^{M}/\left(1-x_{ik}^{M}\right)C_{l}^{M}\right]}=1,$$

and

(11) $$\frac{{\left(x_{kl}^{M}\right)}^{2}\ln\left(\alpha_{kl}C_{ikl}^{M}/x_{kl}^{M}C_{kl}^{M}\right)}{{\left(1-x_{kl}^{M}\right)}^{2}\ln\left[\left(1-\alpha_{kl}\right)C_{ikl}^{M}/\left(1-x_{kl}^{M}\right)C_{i}^{M}\right]}=1,$$

and

(12) $$\frac{{\left(x_{il}^{M}\right)}^{2}\ln\left(\alpha_{il}C_{ikl}^{M}/x_{il}^{M}C_{il}^{M}\right)}{{\left(1-x_{il}^{M}\right)}^{2}\ln\left[\left(1-\alpha_{il}\right)C_{ikl}^{M}/\left(1-x_{il}^{M}\right)C_{k}^{M}\right]}=1,$$

where *M* refers to the micelles, and the other symbols have the same meaning as above.

It is known that, for the ideal mixtures, there are direct relationships between the standard Gibbs free energy of adsorption and the mole fraction of components in the mixed monolayer and between the standard Gibbs free energy of micellization and the mole fraction of components of the mixed micelle. In the case of the non-ideal mixtures of surfactants, the activity of particular components of the mixture in the surface region and micelle should be known. The literature reports different methods for the determination of standard Gibbs free energy of adsorption ($\Delta G_{ads}^{0}$) and micellization ($\Delta G_{mic}^{0}$) [29]. These methods are useful for aqueous solutions of single surfactants. However, they are not quite suitable for aqueous solutions of the multicomponent surfactant mixtures [21]. 

In the aqueous solution of the multicomponent surfactant mixture, three regions can be distinguished: interface (*S*), bulk (*B*), and micellar (M). These regions can be treated as separate phases. The chemical potential of the i-th component of the surfactant mixture in these phases can be expressed as follows [30]:

(13) $$\mu_{i}^{S}=\mu_{i}^{0,S}+RT\ln a_{i}^{S}+\pi_{i}\varpi_{i},$$

and

(14) $$\mu_{i}^{M}=\mu_{i}^{0,M}+RT\ln a_{i}^{M},$$

and

(15) $$\mu_{i}^{B}=\mu_{i}^{*,B}+RT\ln a_{i}^{B},$$

In the equilibrium state, the chemical potential of i-th component is the same. Hence:

(16) $$\mu_{i}^{0,S}-\mu_{i}^{*,B}=RT\ln\frac{a_{i}^{B}}{a_{i}^{S}}-\pi_{i}\varpi_{i},$$

and

(17) $$\mu_{i}^{0,M}-\mu_{i}^{*B}=RT\ln\frac{a_{i}^{B}}{a_{i}^{M}},$$

At constant T and p, $\mu_{i}^{0,S}-\mu_{i}^{*,B}=\Delta G_{ads,i}^{0}$ and $\mu_{i}^{0,M}-\mu_{i}^{*B}=\Delta G_{mic,i}^{0}$. In the case of surfactants, their concentrations in aqueous solution are small, and it can be assumed that $a_{i}^{B}=x_{i}^{B}$ and $x_{i}^{B}≅C_{i}/\omega$. Based on these assumptions the following can be written:

(18) $$\Delta G_{ads,i}^{0}=RT\ln\frac{C_{i}}{\omega x_{i}^{S}f_{i}^{S}}-\pi_{i}\varpi_{i},$$

and

(19) $$\Delta G_{mic,i}^{0}=RT\ln\frac{\mathrm{CMC}\alpha_{i}^{B}}{\omega x_{i}^{M}f_{i}^{M}},$$

where $\varpi_{i}$ is the area occupied by one mole of surfactant, CMC is the critical micelle concentration, and $f_{i}$ is the activity coefficient.

According to the thermodynamic rule, the standard Gibbs free energy of adsorption or micellization of the i-th component of the surfactant mixture ($\Delta G_{i}^{0}$) satisfies the following equation [25,29]:

(20) $$\Delta G_{i}^{0}=\Delta H_{i}^{0}-T\Delta S_{i}^{0},$$

where $\Delta H_{i}^{0}$ is the standard enthalpy of adsorption or micellization, and $\Delta S_{i}^{0}$ is the standard entropy of adsorption or micellization. 

Knowing the values of $\Delta G_{i}^{0}$ at different temperatures and assuming that, in the given temperature range, $\Delta H_{i}^{0}$ is constant, it is possible to determine $\Delta S_{i}^{0}$ from the following equation [25,29]:

(21) $$\frac{d\left(\Delta G_{i}^{0}\right)}{dT}=-\Delta S_{i}^{0},$$

Indeed, knowing the $\Delta G_{i}^{0}$ and $\Delta S_{i}^{0}$ values, the $\Delta H_{i}^{0}$ values can be calculated from Equation (20). The activity coefficients of the i-th component of the surfactant mixture needed for the standard Gibbs free energy of adsorption and micellization determination can be obtained, among other ways, on the basis of intermolecular interactions parameter for the mixed monolayer ($\beta^{\sigma }$) and the micelles ($\beta^{M})$ from the Rosen and Hua equations, which for the two-component mixtures of surfactants have the following forms [27,29]:

(22) $$\ln f_{1}^{S}=\beta^{\sigma }{\left(1-x_{1}^{s}\right)}^{2},$$

and

(23) $$\ln f_{2}^{S}=\beta^{\sigma }{\left(x_{1}^{s}\right)}^{2},$$

and

(24) $$\ln f_{1}^{M}=\beta^{M}{\left(1-x_{1}^{M}\right)}^{2},$$

and

(25) $$\ln f_{2}^{M}=\beta^{M}{\left(x_{1}^{M}\right)}^{2},$$

For calculations of the $\beta^{\sigma }$ and $\beta^{M}$ parameters, Rosen and Hua derived the following equations [27,29]:

(26) $$\beta^{\sigma }=\frac{\ln\left(\alpha_{1}C_{12}^{0}/x_{1}^{S}C_{1}^{0}\right)}{{\left(1-x_{1}^{S}\right)}^{2}},$$

and

(27) $$\beta^{M}=\frac{\ln\left(\alpha_{1}C_{12}^{M}/x_{1}^{M}C_{1}^{M}\right)}{{\left(1-x_{1}^{M}\right)}^{2}},$$

Jańczuk et al. [31,32] showed that there is a relation between the Gibbs free energy of adsorption and/or micellization and the components and parameters of the surface tension of water, as well as the tail and head of surfactant surface tension. They proposed the following equation:

(28) $$\Delta G_{ads}^{0}=\left(\gamma_{T}-\gamma_{TW}\right)A_{T}N+\left(\gamma_{WH1}-\gamma_{WH}\right)A_{H}N,$$

where $\gamma_{T}$ is the tail surface tension; $\gamma_{TW}$, $\gamma_{WH}$, and $\gamma_{WH1}$ are the tail-water, head-water and dehydrated head-water interface tensions, respectively; $A_{T}$ and $A_{H}$ are the contactable areas of the tail and head; N is the Avogadro number. If during the transfer of the surfactant molecule from the bulk phase to the surface region, its head does not dehydrate, then:

(29) $$\Delta G_{ads}^{0}=\left(\gamma_{T}-\gamma_{TW}\right)A_{T}N,$$

The proposed equation for $\Delta G_{mic}^{0}$ has the following form:

(30) $$\Delta G_{mic}^{0}=-N\left[2\gamma_{WT}A_{T}-\left(2\gamma_{WH}-\Delta G_{int}^{EL}\right)A_{H}\right],$$

where $\Delta G_{int}^{EL}$ is the Gibbs free energy of electrostatic interactions.

For nonionic surfactants, Equation (30) assumes the following form:

(31) $$\Delta G_{mic}^{0}=-N\left(2\gamma_{WT}A_{T}-2\gamma_{WH}A_{H}\right),$$

## 3. Results and Discussion

### 3.1. Some Physicochemical Properties of Ber, ELP and RH40

The behavior of surfactants and their mixtures with organic additives in aqueous media depends not only on the types of the chemical groups present in the surfactant molecules and additives but also on their number, as well as their polarity. The sizes of the contactable area of the apolar and polar parts of the surfactants and additives with other molecules and their surface tension components and parameters are responsible for the adsorption and aggregation behaviors of surfactants and their mixtures with organic additives.

The sizes of Ber, ELP, and RH40 molecules were calculated based on the bonds’ lengths, the angles between the bonds, and the distance between the molecules. It was proved that the volume of one molecule each of Ber, ELP, and RH40 can be deduced based on the volumes of cubes into which particular fragments of the molecule can enter. The volumes of Ber, ELP, and RH40 molecules obtained in this way are equal 428.25, 4378.64, and 4825.21 Å^3^, respectively. As follows from these volumes and the Ber, ELP, and RH40 mole weights, the density of these compounds is equal to 1.3042, 0.91947, and 0.9979 g/cm^3^, respectively. Taking into account the molecule size of a given compound obtained in this way, it was possible to establish the contactable area of this molecule with those of water. This area is close to 277.45 Å^2^ for Ber, 3779.9 Å^2^ for ELP, and 4210.2 Å^2^ for RH40. Considering the contactable area of water molecules, which is equal to 10 Å^2^ [33], it can be stated that there can theoretically be contacted 28 water molecules with Ber, 378 with ELP, and 421 with RH40. In turn, one oxyethylene group in the surfactant molecule can be joined with two water molecules by strong hydrogen bonds and three by the weak ones [34,35]. Hence, it can be concluded that, depending on the configuration, the oxyethylene chains in the RH40 molecule can be surrounded at most by about 280 water molecules and ELP by 245. As the contactable area of ELP and RH40 tails is twice as small as the head of their molecules, approximately two times fewer water molecules surround the tails of ELP and RH40 molecules than the heads. The numbers of water molecules surrounding the heads of surfactant molecules make them largely soluble in water. On the other hand, the number of water molecules surrounding the tails of RH40 and ELP molecules is the driving force in their adsorption at the W-A interface and micellization. This driving force depends on the components and parameters of water and surfactants’ surface tension, as well as on the size of the contactable area. The contactable area of the surfactant molecules through the water phase is smaller than that of the whole one. These areas for ELP and RH40 are equal to 951.616 and 1054.582 Å^2^, respectively. In the case of Ber, its contactable area between two molecules is equal to 108.96 Å^2^. This area is smaller than the maximal contactable area between the tails of two ELP and RH40 molecules, which is close to 324.714 Å^2^. 

The surface tension of Ber was determined based on the contact angle $\left(\theta \right)$ measured for water (56.2°), formamide (38.4°), and diiodomethane (46.1°) on pressed berberine using the van Oss et al. concept [18,19,20]. According to this concept and the Young equation, the following can be written:

(32) $$\gamma_{LV}\left(\cos\theta +1\right)=2\left(\sqrt{\gamma_{SV}^{LW}\gamma_{LV}^{LW}}+\sqrt{\gamma_{SV}^{+}\gamma_{LV}^{-}}+\sqrt{\gamma_{SV}^{-}\gamma_{LV}^{+}}\right),$$

Knowing the Lifshitz-van der Waals component ($\gamma_{LV}^{LW})$ of the water, formamide and diiodomethane surface tension, and the electron-acceptor ($\gamma_{LV}^{+}$) and electron-donor ($\gamma_{LV}^{-}$) parameters of this tension [36], it was possible to obtain from Equation (32) the Lifshitz-van der Waals component ($\gamma_{SV}^{LW}$ = 36.42 mN/m) of the Ber surface tension ($\gamma_{SV}$ = 46.52 mN/m), as well as its electron-acceptor ($\gamma_{SV}^{+}$ = 1.56 mN/m) and electron-donor ($\gamma_{SV}^{-}$ = 16.38 mN/m) parameters. From the calculations based on Equation (32), it resulted that the Ber surface tension is not much different from that of Triton X-165 (TX165) if its molecules are positioned with the hydrophilic part toward the air phase [37]. However, the contribution of the LW component to the Ber surface tension is smaller, and that of AB larger than those of TX165 surface tension. The contribution of the LW component to Ber $\gamma_{S}$ is equal to 78%, and it results in the poor solubility of Ber in water. Indeed, the solubility of organic substrates in water depends not only on the LWtoAB ratio and their surface tension but also on the contactable area of the hydrophobic and hydrophilic groups in their molecules. To increase the solubility of Ber in water, it is added to water in the form of an alcohol solution. Probably due to strong interactions between the hydrocarbon groups in the alcohol and in the Ber molecules, the displacement of water molecules surrounding the hydrophobic groups in the Ber molecule, as well as the orientation of the alcohol molecules with the -OH group toward the water phase, enhances the hydrophilic character of the Ber molecules. The constant concentration of ethanol used to dissolve Ber in water, equal to 1 × 10^−3^ mol/dm^3^, practically changes the water surface tension by a value comparable to the accuracy of surface tension measurements. It seems that, in such cases, all thermodynamic considerations dealing with the adsorption and micellization properties of Ber, ELP, and RH40, as well as their mixtures, are reasonable.

In the Ber molecule, in contrast to ELP and RH40, it is difficult to distinguish the hydrophobic (tail) and hydrophilic (head) parts. Therefore, it should be expected that the preferential orientation of Ber molecules at the W-A interface is parallel to this interface. On the other hand, it is possible that the hydrophilic group in the Ber molecule causes its orientation at the W-A interface in the form of an inclined plane. A greater tendency of hydrophobic chains composed of –CH_3_, –CH_2_– or =CH– groups for horizontal orientation than toward reorientation is suggested by the investigators on the basis of thermodynamic considerations of the adsorption processes of different compounds at the W-A interface [38,39,40]. Indeed, this orientation depends largely on the packing of the compound molecules being influenced by the strong intermolecular interactions in the surface region.

Berberine reduces the water surface tension to a small extent (Figure 1), and its maximal reduction corresponds to a Ber concentration close to 1 × 10^−4^ mol/dm^3^. At higher Ber concentrations in aqueous solutions, the surface tension of solution is constant. However, the inflection point on the surface tension isotherm of the berberine aqueous solution cannot be treated as the CMC. The Gibbs surface excess concentration of Ber at the W-A interface calculated from Equation (4) is equal to 1.5 × 10^−6^_,_ 1.48 × 10^−6^_,_ and 1.46 × 10^−6^ mol/dm^3^ at 293, 303, and 313 K, respectively. The minimal area occupied by one Ber molecule is close to the contactable area of berberine at its parallel orientation at the W-A interface. In contrast to berberine, the ELP and RH40 molecules in the surface layer can be oriented perpendicularly and/or at an angle to the W-A interface because their molecules are of an amphiphilic nature. 

It seems that, in the case of ELP and RH40 the directly measured surface tension can be treated as the head surface tension because of very strong hydrophobic interactions between the tails of ELP and RH40 molecules. For this reason, the probability of the orientation of ELP and RH40 molecules at the surfactant-air interface with their heads directed toward the air phase is very high. If so, it is possible to determine the components and parameters of the heads of ELP and RH40 surface tension by measuring their contact angles on the PTFE and PMMA surfaces. It is known that PTFE is a modeling apolar solid, the surface tension of which results from only the Lifshitz-van der Waals intermolecular interactions [18,19,20]. According to Fowkes [41] and van Oss et al. [18,19,20], the equilibrium state of the PTFE-liquid drop-air system fulfills this equation:

(33) $$\gamma_{LV}\left(\cos\theta +1\right)=2\sqrt{\gamma_{SV}^{LW}\gamma_{LV}^{LW}},$$

On the basis of $\gamma_{LV}^{LW}$ and the contact angle on the monopolar PMMA, the electron-acceptor parameter of ELP and RH40 head surface tension can be determined. It should be noted that PMMA is treated as a monopolar solid because its surface tension results from the LW intermolecular interactions [32]. On the other hand, PMMA can also interact with polar liquids by acid-base (AB) forces because the $\gamma^{-}$ parameter of the PMMA surface tension is greater than zero. The contact angle of liquids on the PMMA surface can be expressed by the following equation [18]:

(34) $$\gamma_{LV}\left(\cos\theta +1\right)=2\left(\sqrt{\gamma_{SV}^{LW}\gamma_{LV}^{LW}}+\sqrt{\gamma_{SV}^{-}\gamma_{LV}^{+}}\right),$$

Knowing that $\gamma_{LV}^{AB}=\gamma_{LV}-\gamma_{LV}^{LW}$, it is possible to determine all components and parameters of the ELP and RH40 head surface tension based on the measured values of $\gamma_{LV}$ and θ on the PTFE and PMMA surfaces. These components and parameters determined in this way are similar to those of the head of TX165 surface tension (Table 1). This outcome confirms the assumption that ELP and RH40 molecules are oriented by their heads toward the air phase. In turn, as suggested earlier, the surface tension of the ELP and RH40 heads should be close to those of unsaturated fatty acids [22]. One –OH group in the hydrophobic single chain of ELP and RH40 molecules does not seem to have a significant effect on the value of the tail surface tension. The large size of the head of ELP and RH40 molecules guarantees good solubility of these surfactants in water, and the large size of the tail causes good surface activity and the tendency to form micelles in aqueous media. The maximal Gibbs concentration of ELP and RH40 is greater than for Tritons, and micelle formation takes place at concentrations smaller than that in the case of Tritons [42].

### 3.2. Surface Tension of the Aqueous Solution of ELP, RH40, and ELP + RH40 Mixtures with Ber; Concentration and Composition of Mixed Monolayers at the Water-Air Interface

Taking into account the contribution of the Lifshitz–van der Waals intermolecular interactions in the surface tension of water, Ber, ELP, and RH40, it can be stated that reduction in water surface tension due to their adsorption at the W-A interface depend on only the decrease in the acid-base component of the water surface tension. The LW component of Ber, ELP, and RH40 surface tension is larger than that of water’s surface tension. In the case of the ELP and RH40 tail surface tension, the LW component is only insignificantly larger than that for water (Table 1) [36]. As mentioned above, in Ber molecules, it is difficult to distinguish the tail and head. Therefore, Ber molecules adsorbed in the monolayer at the W-A interface should be oriented parallel to the interface. Hence, the minimal surface tension of the aqueous solution of Ber cannot be smaller than its surface tension (46.52 mN/m) (Table 1). 

**Figure 2:**
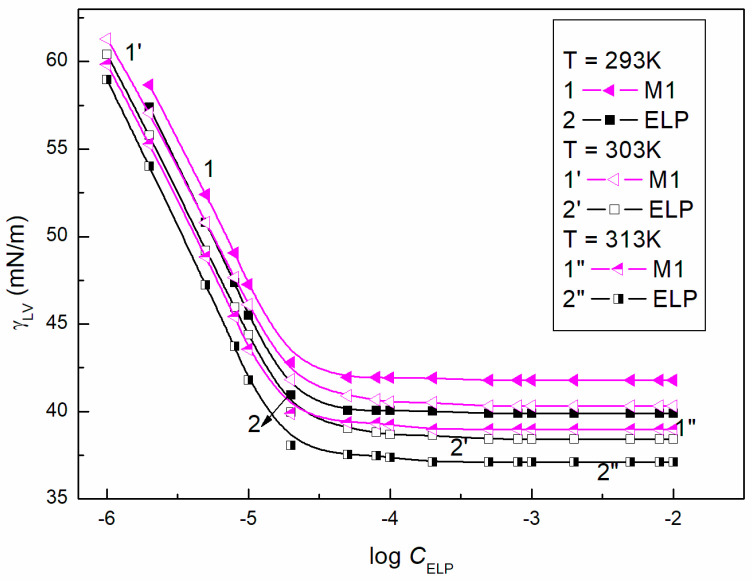
A plot of the surface tension ($\gamma_{LV}$) of M1 aqueous solutions (curves 1, 1′ and 1″) and ELP aqueous solutions [22] (curves 2, 2′ and 2″) vs. the logarithm of ELP concentration (log $C_{\mathrm{ELP}}$) at a constant temperature equal to 293 K (curves 1 and 2), 303 K (curves 1′ and 2′), and 313 K (curves 1″ and 2″).

**Figure 3:**
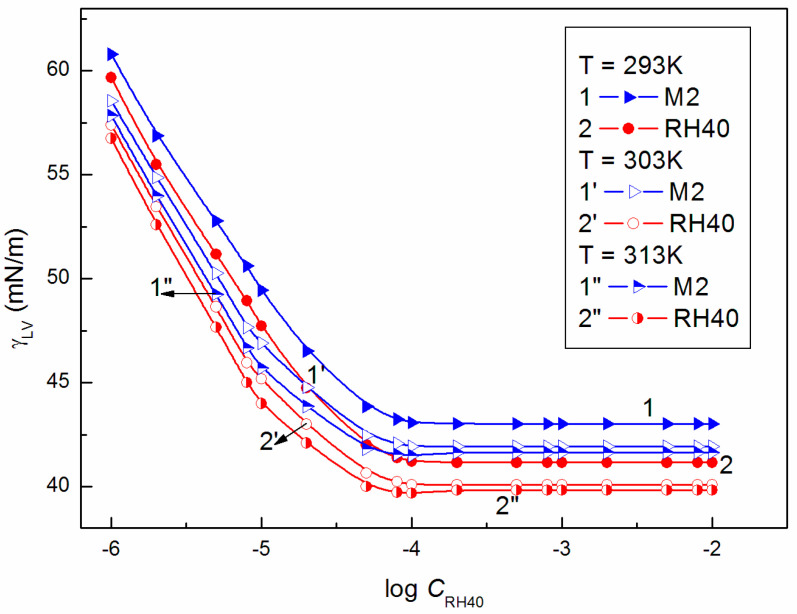
A plot of the surface tension ($\gamma_{LV}$) of M2 mixture aqueous solutions (curves 1, 1′ and 1″) and RH40 aqueous solutions [22] (curves 2, 2′ and 2″) vs. the logarithm of RH40 concentration (log $C_{\mathrm{RH}40}$) at a constant temperature equal to 293 K (curves 1 and 2), 303 K (curves 1′ and 2′), and 313 K (curves 1″ and 2″).

**Figure 4:**
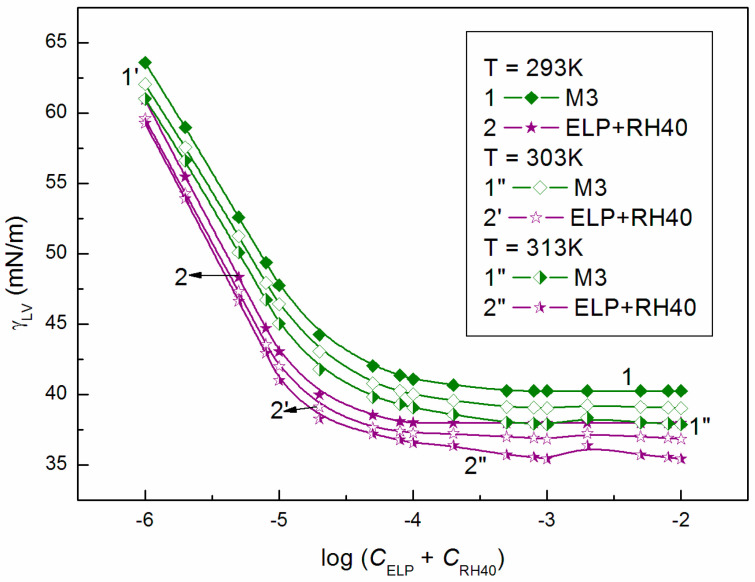
A plot of the surface tension ($\gamma_{LV}$) of M3 mixture aqueous solutions (curves 1, 1′ and 1″) and ELP + RH40 mixture aqueous solutions at a mole fraction of ELP equal to 0.8 [22] (curves 2, 2′ and 2″) vs. the logarithm of the sum of ELP and RH40 concentration (log $\left(\left(C_{\mathrm{ELP}}+C_{\mathrm{RH}40}\right)\right)$ at a constant temperature equal to 293 K (curves 1 and 2), 303 K (curves 1′ and 2′), and 313 K (curves 1″ and 2″).

Assuming that only the surface tension of the ELP and RH40 tail determines the reduction in water surface tension, it can be stated that the minimal surface tension of the aqueous solution should be considerably smaller than that of the Ber aqueous solution. From the differences between the surface tension of Ber and the tail of ELP and RH40, it results that, if the mixed monolayer is formed at the W-A interface from the solution including ELP, RH40, and ELP + RH40 with Ber, the surface tension of the aqueous solution of these mixtures should be greater than that for the solution in the absence of Ber at the same concentrations of ELP, RH40 and ELP + RH40. Removal of the ELP or RH40 molecules from the mixed monolayer at the W-A interface by Ber molecules increases the solution surface tension. This suggestion is confirmed by the isotherms of the surface tension of the aqueous solution of ELP, RH40, and ELP + RH40 mixture in the presence and absence of Ber [22] (Figure 2, Figure 3 and Figure 4). These isotherms have almost the same shape, and at a given temperature, there is almost a linear dependence between the surface tension of the solution and the logarithm of the surfactants or their mixture concentration. The isotherms of the surface tension of the aqueous solution of ELP, RH40, and ELP + RH40 mixture in the presence of Ber, similar to those in it absence [22], can be described by the exponential function of the second order (Figure S1):

(35) $$\gamma_{LV}=y^{0}+A_{1}\exp\left(\frac{-C}{t_{1}}\right)+A_{2}\exp\left(\frac{-C}{t_{2}}\right),$$

where $A_{1}$, $A_{2}$, $t_{1}$, $t_{2}$, and $y^{0}$ are constants.

It was suggested earlier that the constants in Equation (35) are related to the components and parameters of the surface tension of all components of the solution, which are decisive for the interactions between these components [22]. It seems that the constant $y^{0}$ is related to the LW component of the surface tension of all compounds present in the solution and is close to the minimal surface tension of a given solution. This constant changes as a function of temperature almost linearly (Figure S2a). It is likely that the other constants in Equation (35) are closely related to the AB component of the surface tension of water and other compounds present in the aqueous solution. As a matter of fact, the changes in the hydration degree of the Ber, ELP, and RH40 molecules and the distance between these molecules and water can influence on the values of the $A_{1},$ $A_{2}$, $t_{1}$, and $t_{2}$ constants. Presumably for these reasons, the changes in $A_{1},$ $A_{2}$, $t_{1}$, and $t_{2}$ as a function of the temperature are more complicated than the $y^{0}$ constant (Figure S2). The LW components of the Ber, ELP, and RH40 tail surface tension as well as the degree of Ber, tail, and head of ELP and RH40 molecule hydration, are decisive regarding the concentration and composition of the mixed monolayer at the W-A interface.

The concentration of a given component of the surfactant mixture and surfactant mixtures with additives can be indirectly determined, among other ways, using the Gibbs isotherm (Equation (4)) and Frumkin equation (Equation (5)). Unfortunately, in the studied concentration range, which corresponds to the saturated mixed monolayer at the W-A interface, it was possible to determine the maximal concentration of ELP, RH40, and ELP + RH40 mixture in the presence of Ber. It should be noted that the poor solubility of berberine in water will make it impossible to measure the surface tension of the aqueous solutions of the mixture of surfactant and Ber in a broad range of constant surfactant concentration. For this reason, the concentration of the particular surfactant in the single and mixed monolayers at the W-A interface was determined using the Frumkin equation (Equation (5)). To solve this equation in the case of the mixtures, the contribution of their particular components to the reduction in the water surface tension must be known. It was found earlier that the composition of the mixed monolayer can be determined based on the $\gamma_{LV}$ isotherm of the aqueous solution of the single components of the mixture [21,22]. If so, the reduction in the water surface tension by a given mixture component ($\pi_{i}$) can be calculated from Equation (6).

In many cases, it is possible to determine the composition of the binary and ternary mixtures of surfactants using the modified Rosen and Rubingh equations (Equations (8)–(10)) [27,29]. It is known that these equations can be used for the determination of the mole fraction of the mixed monolayer only in limited mixture compositions in the bulk phase. For this reason, it was possible to determine the composition of the mixed monolayer only at small concentrations of surfactants in the M1–M3 mixtures. It was proved that the composition of the mixed monolayer established in this way is close to that determined based on the $\gamma_{LV}$ isotherm of the aqueous solutions of the individual mixture components. The mole fraction of particular components of the M1, M2, and M3 mixtures in the mixed monolayer at the W-A interface is considerably different from that in the bulk phase and depends on the temperature (Figures S3–S5). It is interesting that, at large concentrations of ELP, RH40, or the mixture of ELP + RH40, the mole fraction of Ber in the mixed monolayer is larger than in the bulk phase. This finding indicates that the Ber molecules likely adsorb at the W-A interface together with the surfactant ones. In this case, as a result of strong hydrophobic interactions between Ber and surfactant molecules, the Ber molecules in the mixed monolayer are not oriented parallel and toward the interface but perpendicularly or at a given angle to the interface. This orientation results in an increase in the Ber concentration in the mixed monolayer. In fact, if the berberine concentration in the mixed monolayer is larger than in the bulk phase, then the relation of the mole fraction of surfactants in these phase is reversed. The relation of the composition of the mixed monolayer, including ELP, RH40, and Ber, to that in the bulk phase is slightly different from that for the solutions of mixture M3. In the whole concentration range, the mole fraction of RH40 in the mixed monolayer is larger than in the bulk phase. This relation is consistent with the results for the ELP and RH40 mixture in the absence of Ber [22].

Taking into account the mole fraction of particular mixture components in the mixed monolayer at the W-A interface, the contribution of these components to the reduction in water surface tension was determined using Equation (6). Next, using the Frumkin equation, the concentration of the mixture components in the surface region was calculated. The concentration of the particular components in the mixed monolayer at the W-A interface for all studied mixtures is smaller than those of the individual components in the absence of others (Figures S6–S8). Moreover, the sum of the concentrations of Ber and the surfactant or of Ber and the ELP + RH40 mixture in the mixed surface monolayer is smaller than that of the surfactant or surfactants mixture in the absence of Ber (Figures S6–S8). What causes these phenomena? It is possible, as mentioned above, that the Ber molecules adsorb together with the surfactant ones. The berberine molecules can join the tail of surfactant molecules, decreasing the extent of hydration. As a consequence, a complex of surfactant-Ber is formed with a smaller tendency to adsorb at the W-A interface than the single molecule of ELP or RH40. On the other hand, the adsorbed berberine molecule + surfactant tail complex in the mixed monolayer can be directed toward the water phase, changing its surface tension. Due to the Ber molecule’s structure, it has a positive charge; hence, repulsive electrostatic interactions between the Ber + surfactant tail complexes can appear. These interactions can reduce the packing of the mixed monolayer, decreasing the concentration of surfactant in comparison to its concentration in the absence of Ber.

### 3.3. CMC of ELP + Ber, RH40 + Ber and ELP + RH40 + Ber Mixtures

**Figure 5:**
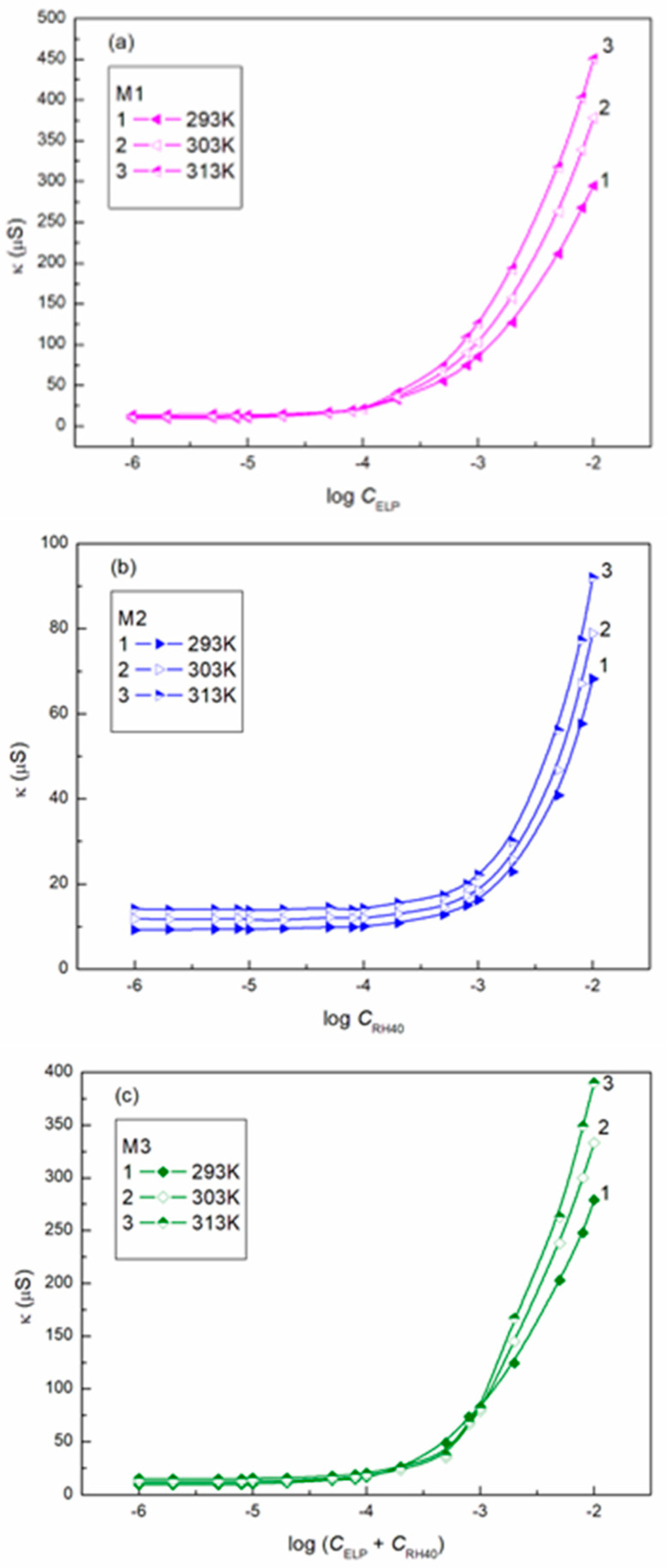
A plot of the specific conductivity (κ) of mixtures M1 (**a**), M2 (**b**), and M3 (**c**) vs. the logarithm of the surfactant or their mixture concentrations at a constant temperature equal to 293 K (curve 1), 303 K (curve 2) and 313 K (curve 3).

**Figure 6:**
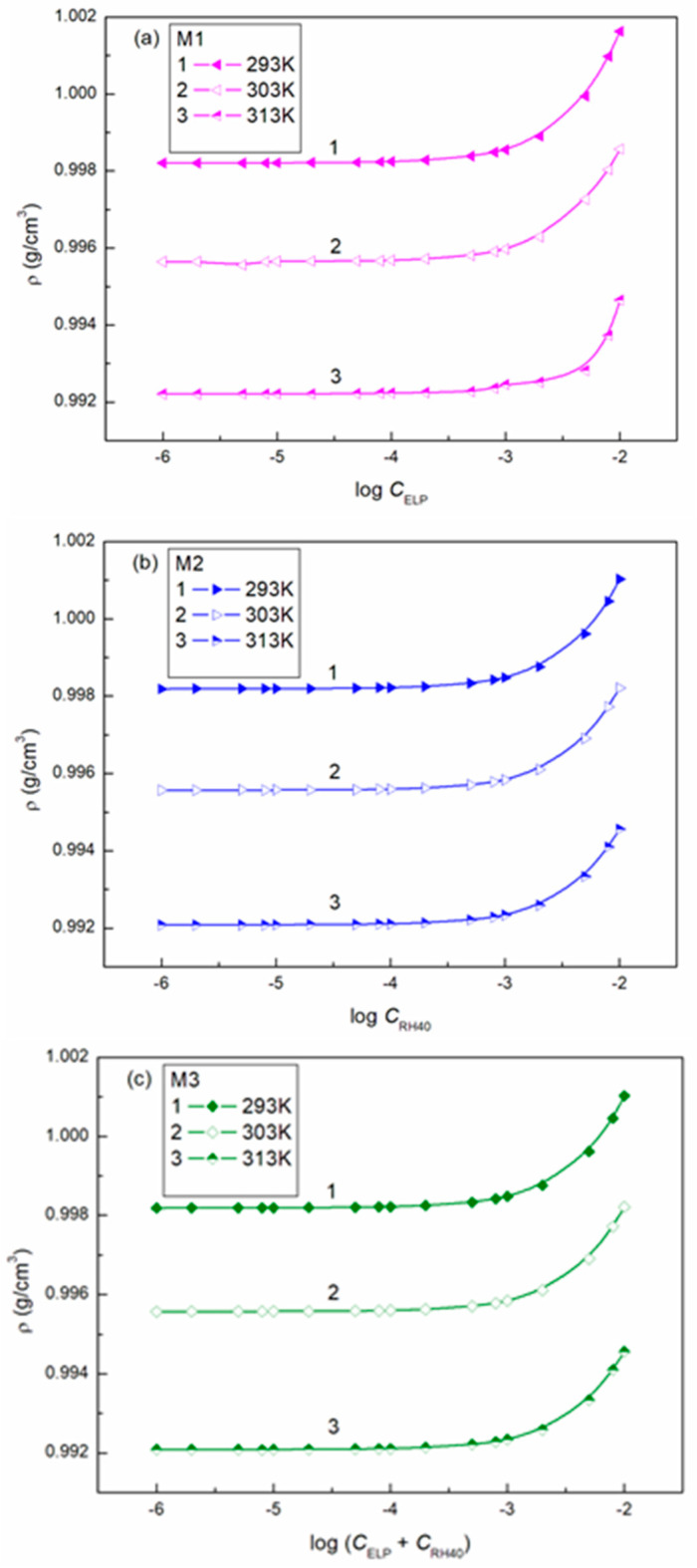
A plot of the density (ρ) of mixtures M1 (**a**), M2 (**b**) and M3 (**c**) vs. the logarithm of the surfactant or their mixture concentration at a constant temperature equal to 293 K (curve 1), 303 K (curve 2), and 313 K (curve 3).

**Figure 7:**
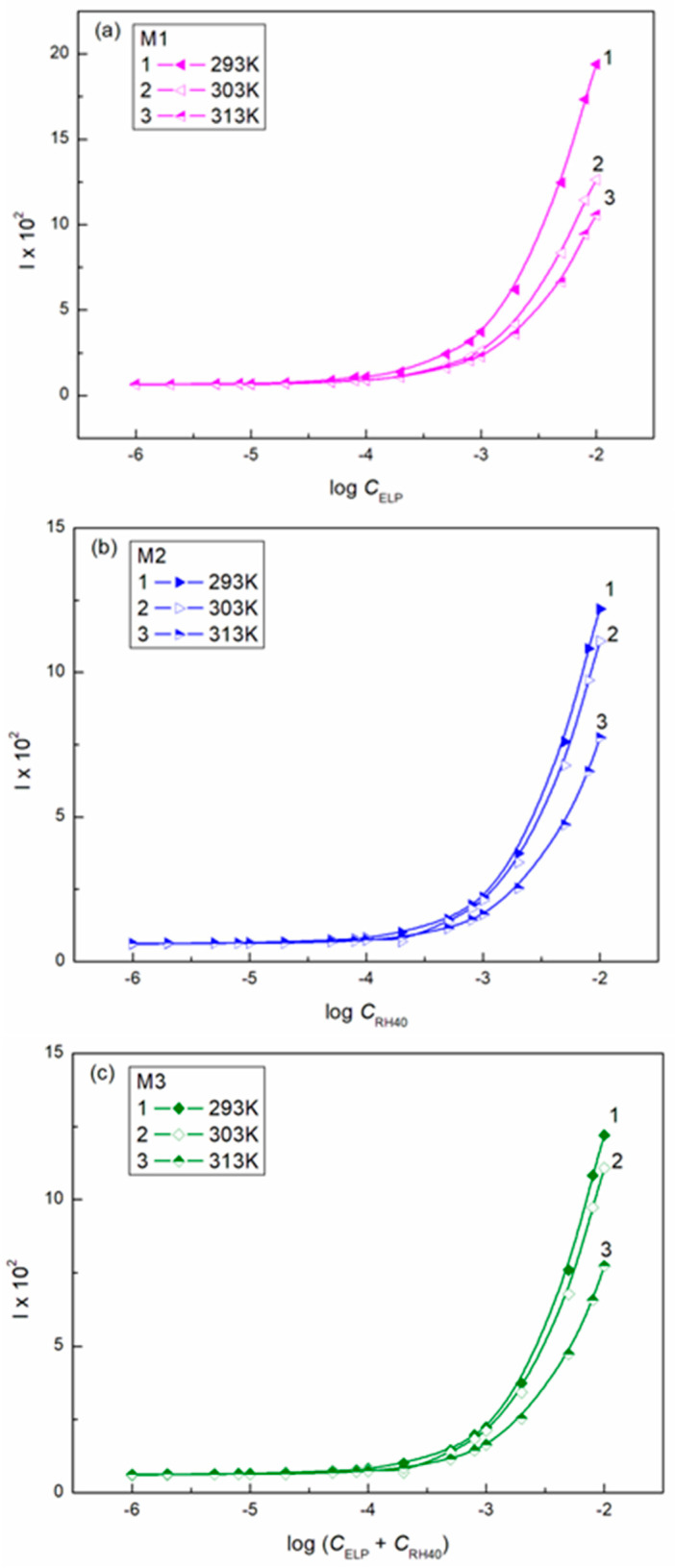
A plot of the fluorescence intensity (I) of mixtures M1 (**a**), M2 (**b**), and M3 (**c**) vs. the logarithm of the surfactant or their mixture concentration at a constant temperature equal to 293 K (curve 1), 303 K (curve 2), and 313 K (curve 3).

**Figure 8:**
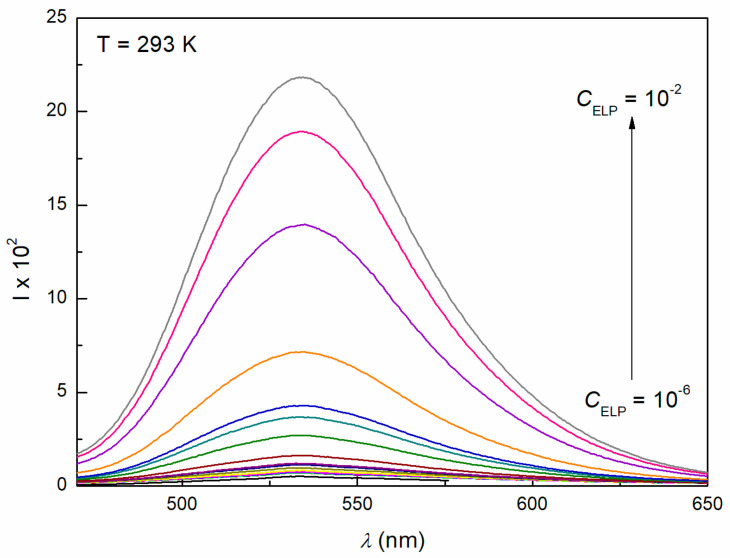
Fluorescence emission spectra of mixture M1 at *T* = 293 K.

The ability of surfactants to form aggregates in aqueous solution is one of their characteristic and a very important property. The micellar phase plays a very important role, among others, because of its solubilization properties. We were interested in whether Ber is present in the micelles formed by ELP, RH40, and their mixture. To consider this problem, it was first necessary to determine the CMC and the composition of the mixed micelles. In the literature, there are many methods for CMC determination, for example, based on the isotherms of the surface tension, density, and conductivity and using spectroscopic methods. Formation of micelles in aqueous media is reflected by the inflection point appearing on these isotherms [29]. However, it should be remembered that each method can be sensitive to a different size and shape of aggregates formed by surfactants. The CMC values of surfactant + Ber mixtures (M1–M3) determined from the $\gamma_{LV}$ isotherms (Figure 2, Figure 3 and Figure 4), as well as conductivity (Figure 5), density (Figure 6) measurements, and fluorescence emission spectra (Figure 7 and Figure 8 as an example), are different even for the same mixture (Table 2). The CMC values of the surfactants or their mixture in the presence of Ber are higher than those in its absence.

Formation of micelles in aqueous solution by surfactants at their given concentrations is due to hydrophobic interactions between the tails of surfactants through the water phase. The values of these interactions are positive, in contrast to the interactions of the surfactant head, which are negative independent of whether micelles are formed by nonionic or ionic surfactants. According to Equations (30) and (31) [31,32], the power of the hydrophobic interactions depends on the tail-water interface tension and the contactable area of the tail. The interface tension can be calculated from, among others, the van Oss et al. [18,19,20] concept. From this concept it follows:

(36) $$\gamma_{ij}=\gamma_{i}+\gamma_{j}-2\left(\sqrt{\gamma_{i}^{LW}\gamma_{j}^{LW}}+\sqrt{\gamma_{i}^{+}\gamma_{i}^{-}}+\sqrt{\gamma_{i}^{-}\gamma_{j}^{+}}\right),$$

If the surface tension of at least one of the phases being in contact results from only the LW intermolecular interactions, then Equation (36) takes the following form:

(37) $$\gamma_{ij}=\gamma_{i}+\gamma_{j}-2\sqrt{\gamma_{i}^{LW}\gamma_{j}^{LW}},$$

Taking into account Equations (36) and (37) the components and parameters of berberine, water, and surfactant tail surface tension (Table 1), the values of the water tails of surfactants, water-Ber, and Ber-surfactant tail interface tension were calculated. The obtained values of the water-surfactant tail, water-Ber, and Ber-surfactant tail interface tension are close to 46.0, 6.0, and 10.7 mN/m, respectively. Based on these values, it was possible to determine the adhesion work of the surfactant tail to the surfactant tail, the surfactant tail to Ber, and Ber to the surfactant tail through the water phase. The adhesion work of the surfactant tail to the surfactant tail through the water phase is equal to $2\gamma_{WT}$ = 92 mJ/m^2^ and that of Ber to Ber through the water phase to $\gamma_{WB}$ = 12 mJ/m^2^. In the case of the Ber-water-surfactant tail system, the adhesion work ($W_{a}^{BT}$) was calculated from the following equation [25]:

(38) $$W_{a}^{BT}=\gamma_{WT}+\gamma_{WB}-\gamma_{BT},$$

The value of $W_{a}^{BT}$ calculated from Equation (38) is equal to 41.3 mJ/m^2^.

As the surfactant head-water interface tension of both surfactants is negative and close to −18 mJ/m^2^, their total adhesion work through the water phase is close to 56 mJ/m^2^. Thus, there is not a large difference in the tendency to contact the surfactant with the surfactant molecule and the surfactant with the Ber molecule through the water phase. However, the probability of the binding of Ber+ surfactant complexes through the water phase is smaller than that of surfactant molecules. Presumably for this reason, the CMC of ELP, RH40, and ELP + RH40 mixtures is higher in the presence of Ber than in its absence (Table 2). Based on these facts, it can be assumed that the micellization process of the surfactant mixture occurs as a result of connecting not only surfactant molecules but also surfactants with Ber molecules. Thus, it seems that the presence of Ber in the micelles of ELP, RH40, and their mixture is not due to the adsorption of Ber molecules on the micelles and its penetration into the micelles but rather by common aggregation. For mixed micelles of ELP + Ber (M1) and RH40 + Ber (M2), it is difficult to determine the mole fraction of surfactant and Ber in the micelles based on their CMC values. This possibility was based on the modified concept of Hua and Rosen (Equations (10)–(12)) [27,29]. The calculated mole fractions of ELP, RH40, and Ber showed that the mole fraction of Ber does not differ significantly from 0.5. This outcome indicated that the interactions of berberine + surfactant complexes through the water phase play a major role in the aggregation process. Indeed, the mole fraction of particular components of ELP + RH40 + Ber mixtures (M3) changes to a small extent as a function of temperature (Table 3). The change in the mole fractions of the mixture components, as well as the CMC value itself as a function of temperature, results, on the one hand, from the change in kinetic energy and, on the other hand, from the change in hydration degree, especially of the surfactant heads. A change in the configuration of the surfactant molecules can also have an effect.

The influence of these factors on the composition and size of micelles should be reflected in the parameter of intermolecular interactions. This parameter can be determined, among other ways, from Equation (27) [27,29]. Since Ber does not form micelles on its own, it was not possible to determine the interactions parameter between Ber and surfactants molecules in the micelles formed in the ELP + Ber and RH40 + Ber systems (M1 and M2). Considering the complexes of ELP + Ber and RH40 + Ber as individual compounds, it was possible to determine the interactions parameter ($\beta^{M}$) of Ber with ELP and RH40 in the mixed micelles of mixture M3 using the Hua and Rosen equation (Equation (27)). As follows from Table 3, the $\beta^{M}$ parameter, calculated based on CMC determined from the surface tension measurements, is negative for the RH40 + Ber complex at each temperature but for ELP + Ber complex only at *T* = 293 K. A similar situation can be observed for the values of the excess Gibbs energy of micelle formation per mole of the surfactant mixture ($G^{M}$). In fact, the values of $\beta^{M}$ depend on the temperature for both complexes. The negative $\beta^{M}$ parameter suggests that there is synergism in the CMC. Unfortunately, this suggestion cannot be confirmed by the second condition for the existence of synergy for the abovementioned reason. It should also be noted that the values of the activity coefficient of ELP in the mixed micelles (Table 3) decrease, but for RH40, they increase with the *T* increase.

### 3.4. Thermodynamic Parameters of the Adsorption and Micellization

The standard Gibbs free energy, standard enthalpy, and entropy are useful to determine the tendency of the surfactants to adsorb at different interfaces and to form micelles, as well as the reason for this tendency. In the literature, there are many different methods used to determine the standard Gibbs free energy of adsorption ($\Delta G_{ads}^{0}$) and micellization ($\Delta G_{mic}^{0}$), the standard enthalpy of adsorption $\Delta (H_{ads}^{0}$) and micellization ($\Delta H_{mic}^{0}$), and the standard entropy of adsorption ($\Delta S_{ads}^{0})$ and micellization $\left(\Delta S_{mic}^{0}\right)$ of the single compounds. Regarding cases in which the mixed monolayer at the interfaces and mixed micelles is formed, it is difficult to find in the literature methods strictly connected with thermodynamics rules. Therefore, the criteria that should be fulfilled to calculate the real values of the thermodynamic parameters of adsorption and micellization are presented above. Unfortunately, it was impossible to determine the activity coefficients of the components in the mixed monolayer at the W-A interface based on the Hua and Rosen concept. Therefore, the $\Delta G_{ads}^{0}$ values for Ber, ELP, and RH40 in the M1-M3 mixture was calculated from Equation (18), assuming the activity coefficients to be close to unity. The obtained results showed that, in the range of surfactants concentration at which they are present in the solution in the monomeric form, the $\Delta G_{ads}^{0}$ values are almost constant (Table 4). At the surfactants concentration higher than CMC the $\Delta G_{ads}^{0}$ values increase as a function of concentration. However, it should be mentioned that the surfactant molecules adsorb at the water-air interface only in the monomeric form. At surfactant concentrations higher than the CMC, their concentration in the monomeric form is constant. For this reason, the $\Delta G_{ads}^{0}$ values are not real.

To compare the $\Delta G_{ads}^{0}$ values obtained from Equation (18) to those determined using other methods, the calculations of $\Delta G_{ads}^{0}$ were performed using the Langmuir equation modified by de Boer [29,43]. This equation has the following form:

(39) $$\frac{A_{i}^{0}}{A_{i}-A_{i}^{0}}\exp\frac{A_{i}^{0}}{A_{i}-A_{i}^{0}}=\frac{C_{i}}{\omega }\exp\left(\frac{-\Delta G_{ads,i}^{0}}{RT}\right),$$

where $A_{i}$ and $A_{i}^{0}$ are the areas occupied by one molecule of the i-th component of the mixture in the mixed monolayer and the limiting one.

The $\Delta G_{ads}^{0}$ values calculated from Equation (39) based on the concentration of particular components in the mixed monolayer at the W-A interface, determined from the Frumkin equation (Figure S9) in the range of surfactant concentrations smaller than CMC, are similar to those obtained from Equation (18) (Table 4, Figures S9–S11). From the obtained $\Delta G_{ads}^{0}$ values, it can be stated that the Ber presence decreases the ELP and RH40 tendency to adsorb at the W-A interface. This outcome confirms our suggestion that the surfactant + Ber molecule complexes can be adsorbed at this interface. As mentioned above, there are some differences in the hydration degree of the molecule complexes in comparison to the individual ones. In such cases, there is also a reduction in the difference between the tail-water and tail-air interface tension, which according to Equation (28) decreases $\Delta G_{ads}^{0}$. The standard enthalpy of adsorption of particular components of the studied mixtures, determined based on Equations (20) and (21) in most cases, has a small absolute value except for the standard enthalpy value for RH40 adsorption at the W-A interface from the aqueous solution of mixture M3 (Table 4). The small absolute values of $\Delta H_{ads}^{0}$ indicate that our suggestion about the adsorption of surfactant + berberine complexes is the most probable.

In many cases, the absolute values of the standard enthalpy of micellization are greater than those of the standard enthalpy of adsorption (Table 4 and Table 5). In most studied systems, the $\Delta H_{mic}^{0}$ is positive, indicating that some bonds are broken during the micellization process. In fact, it should refer to hydrogen bonds. There are some differences between the behavior of ELP and RH40. In solutions including RH40, the greatest changes of the standard enthalpy are observed in both adsorption and micellization processes compared to in the absence of RH40.

## 4. Materials and Methods

Kolliphor^®^ ELP (ELP) (Cremophor^®^ELP, Polyoxyl 35 hydrogenated castor oil, polyoxyl-35 castor oil), Kolliphor^®^ RH 40 (RH40) (Cremophor^®^ RH 40, macrogolglycerol hydroxystearate, PEG-40 castor oil, polyoxyl 40 hydrogenated castor oil) (Sigma-Aldrich (St. Louis, MO, USA) and berberine chloride (Ber) (Alfa Aesar, Kandel, Germany) were used without further purification. The doubly distilled and deionized water used for the preparation of the aqueous solutions of ELP + Ber (M1), RH40 + Ber (M2), and ELP + RH40 + Ber mixture (M3, the mole fraction of ELP (α) in the bulk phase equal to 0.8) was obtained from a Destamat Bi18E distiller (Inkom Instruments, Warsaw, Poland). The surfactant solution concentration was from 1 × 10^−6^ to 1 × 10^−2^ mol/dm^3^, and the Ber concentration in the surfactant solutions was equal to 1 × 10^−4^ mol/dm^3^. Because Ber is poorly soluble in water, it was introduced into the aqueous solutions in the form of an ethanolic solution, similar to other research [44,45,46]. The concentration of ethanol in the aqueous solution was constant and equal to 1 × 10^−3^ mol/dm^3^.

The surface tension (*γ*_LV_) measurements of the aqueous solutions of the M1, M2, and M3 mixtures were performed at temperatures of 293, 303, and 313 K using a Krüss K100 tensiometer (Krüss, Hamgurg, Germany), which was calibrated before the measurements, according to the platinum ring tensiometer method (du Nouy’s method). The calibration was performed at 293 K using water and methanol, the surface tension values of which at this temperature were equal to 72.8 and 22.5 mN/m, respectively. The surface tension measurements for each concentration and composition of the studied solutions were repeated at least ten times. The standard deviation of the results obtained from the measurements was ±0.1 mN/m, and the uncertainty was in the range of 0.3% to 0.9%.

Measurements of the advancing contact angle (*θ*) were performed using the sessile drop method with a DSA30 measuring system (Krüss, Germany) in a temperature-controlled chamber. For *θ* measurements on pressed Ber water (Destamat Bi18E), formamide (>99.5%, Sigma-Aldrich (St. Louis, MO, USA) and diiodomethane (>99%, Sigma-Aldrich, St. Louis, MO, USA) were used. As previous studies showed, the value of the contact angle depends on the difference between the interface pressure and the hydrostatic pressure of the drop [47]. Therefore, in the contact angle measurements, the droplet sizes were different for diiodomethane, formamide, and water and equaled 4, 5, and 6 cm^3^, respectively. Ten drops for each studied system were used, and the standard deviation was in the range of 1 to 1.5°.

The conductivity measurements were performed using a Mettler Toledo™ Seven Multi with accuracy of ±0.5%.

The density of the studied solutions was measured with a U-tube densitometer (DMA 5000 Anton Paar). The precision of the density and temperature measurements given by the manufacturer is ±0.000005 g cm^−3^ and ±0.001 K. The uncertainty was calculated to be 0.01%. The densitometer was calibrated regularly with distilled and deionized water.

Steady-state fluorescence measurements were performed using a Hitachi F-2700 Fluorescence spectrometer. Fluorescence excitation was determined at 450 nm, and the emission spectra were recorded in the range of 350–650 nm at a scan speed of 300 nm/min. The excitation and emission slit widths were 2.5 nm.

## 5. Conclusions

The measurements and discussion of the obtained results based on the thermodynamic rules allow for drawing interesting conclusions. Berberine (Ber) is a bipolar compound, and its surface tension results from the Lifshitz–van der Waals and acid-base components. However, the contribution of the Lifshitz–van der Waals component is considerably larger than that of the acid-base one, indicating that Ber has poor solubility in water. In turn, the electron-acceptor-parameter of the acid-base component is considerably smaller than that of the electron-donor one.

Berberine reduces the water surface tension to a small extent, and its maximal Gibbs surface excess concentration is considerably smaller than those of ELP and RH40.

The minimal area occupied by one Ber molecule is close to its contactable area at a parallel orientation toward the water-air interface.

The components and parameters of the ELP and RH40 surface tension at the orientation of their molecules towards the air phase can be determined from their contact angle on the PTFE and PMMA surfaces. These components and parameters are similar to those of the TX165 surface tension at its orientation by the hydrophilic part towards the air phase.

The molecules of ELP and RH40 adsorbed at the water-air interface reduced only the LW component of the water surface tension.

The contribution of oxyethylene groups to the surface tension of ELP and RH40 is similar to that of Triton X-165.

The surface tension isotherm of the aqueous solution of Ber mixture with ELP, RH40, and ELP + RH40 can be described by the exponential function of the second order.

The LW components of the Ber, ELP, and RH40 tail surface tension, as well as the hydration degree of Ber molecules and the tail and head of ELP and RH40 molecules, are decisive regarding the concentration and composition of the mixed monolayer at the W-A interface.

The composition of the mixed monolayer at the W-A interface, determined based on the surface tension isotherm of aqueous solutions of Ber, ELP, and RH40, is close to that obtained from the Rosen and Rubingh equations. This composition allows for determining the surface concentration of particular components in the mixed monolayer, as well as their concentrations in the mixed monolayer at the W-A interface using the Frumkin equation.

At large concentrations of ELP, RH40, or the mixture of ELP + RH40, the mole fraction of Ber in the mixed monolayer is larger than in the bulk phase.

The concentration of the particular components in the mixed monolayer at the W-A interface for all studied mixtures is smaller than those of the individual components in the absence others.

The sum of the concentrations of Ber and the surfactant or of Ber and the ELP + RH40 mixture in the mixed surface monolayer is smaller than that of the surfactant or surfactant mixture in the absence of Ber.

The CMC values of the surfactant + Ber mixtures, determined from the surface tension isotherms, conductivity, density, and fluorescence emission spectroscopy, are different even for the same mixture.

The concentration of Ber in the mixed micelles is higher than in the bulk phase. This outcome indicates that the tendency of Ber toward solubilization in the micelles of ELP, RH40, and ELP + RH40 is greater than its tendency to adsorb at the water-air interface.

Using our thermodynamic considerations and the Hua and Rosen concept, it was possible to determine the standard thermodynamic parameters of adsorption and aggregation.

## Figures and Tables

**Table 1:** The values of the contact angles of water $\left(\theta_{W}\right)$, formamide $\left(\theta_{F}\right)$, and diidomethane $\left(\theta_{D}\right)$ on the pressed Ber; the contact angles (θ) of ELP and RH40 on PTFE and PMMA surfaces; and the components and parameters of the surface tension of water, Ber, ELP, ELP/RH40/TX165 head, PTFE and PMMA.

	$\theta_{W}$	$\theta_{F}$	$\theta_{D}$	
Ber	56.2	38.4	46.1	
	θ PTFE		θ PMMA	
ELP	74		23	
RH40	75		25	
	$\gamma_{LV}^{LW}$	$\gamma_{LV}^{+}$	$\gamma_{LV}^{-}$	$\gamma_{LV}$
Ber	36.42	1.556	16.38	46.52
ELP head	27.51	0.460	48.95	37.00
RH40 head	27.38	0.565	44.42	37.40
Water	26.85	22.975	22.975	72.80
TX165 head	27.70	0.33	50.20	35.84
PTFE	20.24	-	-	20.24
PMMA	41.28	-	7.28	41.28

**Table 2:** The values of the CMC (mol/dm^3^) of the aqueous solutions M1, M2, and M3 mixtures as, well as ELP, RH40 and their mixture at *T* = 293, 303, and 313 K, determined from the surface tension isotherms ($\gamma_{LV}$ = f(log *C*)), conductivity (κ = f(1/*C*)), and density (ρ = f(1/*C*)) measurements, as well as fluorescence emission spectra (*I* = f(1/*C*)). The values of CMC for ELP, RH40, and their mixture, determined from surface tension measurements, are taken from Ref. [22].

	*T* (K)	CMC from $\gamma_{LV}$	CMC from κ	CMC from ρ	CMC from *I*
ELP + Ber (M1)	293	2.11 × 10^−5^	3.87 × 10^−4^	5.92 × 10^−4^	
	303	2.78 × 10^−5^	4.02 × 10^−4^	5.40 × 10^−4^	
	313	2.72 × 10^−5^	4.67 × 10^−4^	5.05 × 10^−4^	
ELP	293	2.14 × 10^−5^		5.13 × 10^−4^	6.03 × 10^−4^
	303	2.03 × 10^−5^		7.78 × 10^−4^	4.16 × 10^−4^
	313	1.91 × 10^−5^		5.32 × 10^−4^	3.71 × 10^−4^
RH40 + Ber (M2)	293	4.24 × 10^−5^	7.31 × 10^−4^	4.97 × 10^−4^	
	303	5.62 × 10^−5^	7.70 × 10^−4^	4.67 × 10^−4^	
	313	5.05 × 10^−5^	4.46 × 10^−4^	4.16 × 10^−4^	
RH40	293	6.64 × 10^−5^		6.03 × 10^−4^	4.97 × 10^−4^
	303	2.50 × 10^−5^		5.77 × 10^−4^	4.67 × 10^−4^
	313	2.12 × 10^−5^		5.22 × 10^−4^	4.42 × 10^−4^
ELP + RH40 + Ber (M3)	293	3.13 × 10^−5^	3.81 × 10^−4^	4.74 × 10^−4^	
	303	2.81 × 10^−5^	4.23 × 10^−4^	4.33 × 10^−4^	
	313	2.80 × 10^−5^	4.74 × 10^−4^	4.16 × 10^−4^	
ELP + RH40	293	1.92 × 10^−5^		8.76 × 10^−4^	4.89 × 10^−4^
	303	1.84 × 10^−5^		7.21 × 10^−4^	4.40 × 10^−4^
	313	1.70 × 10^−5^		7.03 × 10^−4^	3.90 × 10^−4^

**Table 3:** The values of the fraction of area occupied by a component in the micelles in mixture M3 ($x^{M}$), intermolecular interactions parameters for mixed micelle ($\beta^{M})$, activity coefficient ($f^{M}$), and the excess Gibbs energy of micelle formation per mole of the surfactant mixture ($G^{M}$) calculated from the Rosen and Hua concept (Equations (10)–(12), (24), (25) and (27)) at *T* = 293, 303, and 313 K (1—ELP, 2—RH40, 3—Ber).

	*T* = 293 K	*T* = 303 K	*T* = 313 K
CMC	3.13 × 10^−5^	2.81 × 10^−5^	2.80 × 10^−5^
*C*_1_	2.50 × 10^−5^	2.25 × 10^−5^	2.24 × 10^−5^
*C*_2_	6.26 × 10^−6^	5.63 × 10^−6^	5.60 × 10^−6^
*C*_3_	1 × 10^−4^	1 × 10^−4^	1 × 10^−4^
γ CMC	40.27	39.17	37.92
$x_{1}^{M}$	0.3954	0.4568	0.4508
$x_{2}^{M}$	0.2313	0.0418	0.0024
$x_{3}^{M}$	0.3733	0.5014	0.5467
$\beta_{\left(13\right)-2}^{M}$	−5.0834	0.3762	1.0993
$\beta_{\left(23\right)-1}^{M}$	−0.4159	−1.5256	−1.1598
$f_{13}^{M}$	0.7619	1.0007	1.0007
$f_{2}^{M}$	0.0496	1.4125	2.8469
$f_{23}^{M}$	0.9371	0.7273	0.7900
$f_{1}^{M}$	0.8590	0.6376	0.7048
$G_{\left(13\right)-2}^{M}$	−2.2018	0.0380	0.0682
$G_{\left(23\right)-1}^{M}$	−0.2422	−0.9536	−0.7472

**Table 4:** Thermodynamic parameters of the adsorption process of ELP, RH40 and Ber (kJ/mol).

	*T* = 293 K			*T* = 303 K			*T* = 313 K		
	$\Delta G_{ads}^{0}$	$\Delta H_{ads}^{0}$	$T\Delta S_{ads}^{0}$	$\Delta G_{ads}^{0}$	$\Delta H_{ads}^{0}$	$T\Delta S_{ads}^{0}$	$\Delta G_{ads}^{0}$	$\Delta H_{ads}^{0}$	$T\Delta S_{ads}^{0}$
ELP	−46.91	−1.64	45.27	−47.97	−1.16	46.81	−50.00	−1.64	48.36
ELP from M1	−44.62	−0.23	44.39	−46.16	−0.26	45.90	−47.65	−0.23	47.42
ELP from M3	−43.64	2.65	46.29	−45.25	2.62	47.87	−46.80	2.65	49.45
RH40	−48,12	4.62	52.74	−50.11	4.43	54.54	−51.72	4.62	56.34
RH40 from M2	−45.69	2.54	48.23	−47.57	2.30	49.87	−48.98	2.54	51.52
RH40 from M3	−44.45	−8.85	35.6	−45.72	−8.91	36.81	46.88	84.91	38.03
Ber	−32.00	−1.97	30.03	−33.10	−2.04	31.06	−34.05	−1.97	32.08
Ber from M1	−31.63	−1.89	29.74	−32.62	−1.87	30.75	−43.66	−11.89	31.77
Ber from M2	−31.42	−3.15	28.27	−32.32	−3.08	29.24	−33.45	−3.25	30.20
Ber from M3	−31.02	−1.28	29.74	−32.02	−1.27	30.75	−32.95	−1.18	31.77

**Table 5:** Thermodynamic parameters of the micellization process of ELP, RH40, and Ber (kJ/mol).

	*T* = 293 K			*T* = 303 K			*T* = 313 K		
	$\Delta G_{mic}^{0}$	$\Delta H_{mic}^{0}$	$T\Delta S_{mic}^{0}$	$\Delta G_{mic}^{0}$	$\Delta H_{mic}^{0}$	$T\Delta S_{mic}^{0}$	$\Delta G_{mic}^{0}$	$\Delta H_{mic}^{0}$	$T\Delta S_{mic}^{0}$
ELP	−35.97	4.17	40.14	−37.33	4.18	41.51	−38.71	4.17	42.88
ELP from ELP + RH40	−35.81	6.68	42.49	−37.33	6.61	43.94	−38.71	6.68	45.39
ELP from M3	−32.96	1.47	34.43	−33.96	1.64	35.60	−35.31	1.47	36.78
RH40	−33.21	8.98	42.19	−36.80	6.83	43.63	−38.44	6.63	45.07
RH40 from RH40 + ELP	−36.19	−3.23	32.96	−36.80	−2.71	34.09	−38.44	−3.23	35.21
RH40 from M3	−31.28	16.92	48.20	39.88	89.72	49.84	−43.71	7.78	51.49
Ber from M3	−29.15	4.55	33.70	−31.57	3.28	34.85	−32.72	3.28	36.00

## Data Availability

The data presented in this study are available in Supplementary Materials.

## Supplementary Materials

The following supporting information can be downloaded at: https://www.mdpi.com/article/10.3390/molecules28073115/s1, Figure S1: A plot of the surface tension ($\gamma_{LV}$) of M1 (a), M2 (b), and M3 (c) aqueous solutions vs. the logarithms of ELP ($\log C_{\mathrm{ELP}}$) and RH40 ($\log C_{\mathrm{RH}40}$) and that of the sum of their concentrations $\left(\log\left(C_{\mathrm{ELP}}+C_{\mathrm{RH}40}\right)\right)$ at *T* = 293 K, 303 K, and 313 K. Figure S2: A plot of the constant $y^{0}$ (a), $A_{1}$ (b), $A_{2}$ (c), $t_{1}$ (d), and $t_{2}$ (e) in Equation (35) vs. the temperature (*T*). Figure S3: A plot of the mole fraction of ELP and Ber in M1 in the surface layer and in the bulk phase vs. the logarithm of ELP concentration ($\log C_{\mathrm{ELP}}$) at *T* = 293 K, 303 K, and 313 K. Figure S4: A plot of the mole fraction of RH40 and Ber in M2 in the surface layer and in the bulk phase vs. the logarithm of RH40 concentration ($\log C_{\mathrm{RH}40}$) at *T* = 293 K, 303 K, and 313 K. Figure S5: A plot of the mole fraction of ELP, RH40, and Ber in M3 in the surface layer and in the bulk phase vs. the logarithm of the sum of ELP and RH40 concentrations ($\log(C_{\mathrm{ELP}}+C_{\mathrm{RH}40}$) at *T* = 293 K, 303 K, and 313 K. Figure S6: A plot of the surface concentration (Γ) calculated from Equation (5) vs. the logarithm of ELP concentration ($\log C_{\mathrm{ELP}}$) at *T* = 293 K, 303 K, and 313 K. Figure S7: A plot of the surface concentration (Γ) calculated from Equation (5) vs. the logarithm of RH40 concentration ($\log C_{\mathrm{RH}40}$) at *T* = 293 K, 303 K, and 313 K. Figure S8: A plot of the surface concentration (Γ) of the M3 mixture components calculated from Equation (5) vs. the logarithm of the sum of ELP and RH40 concentrations ($\log(C_{\mathrm{ELP}}+C_{\mathrm{RH}40}$) at *T* = 293 K, 303 K, and 313 K. Figure S9: A plot of the Gibbs standard free energy of adsorption ($\Delta G_{ads}^{0}$) calculated from Equation (39) vs. the logarithm of ELP concentration ($\log C_{\mathrm{ELP}}$) at *T* = 293 K, 303 K, and 313 K. Figure S10: A plot of the Gibbs standard free energy of adsorption ($\Delta G_{ads}^{0}$) calculated from Equation (39) vs. the logarithm of RH40 concentration ($\log C_{\mathrm{RH}40}$) at *T* = 293 K, 303 K, and 313 K. Figure S11: A plot of the Gibbs standard free energy of adsorption ($\Delta G_{ads}^{0}$) of the M3 mixture components calculated from Equation (39) vs. the logarithm of the sum of ELP and RH40 concentrations $\left(\log\left(C_{\mathrm{ELP}}+C_{\mathrm{RH}40}\right)\right)$ at *T* = 293 K, 303 K and 313 K.

## Sample Availability

Samples of the compounds are not available from the authors.
