# Supplementary material for: Thermodynamic Parameters of Berberine with Kolliphor Mixtures Adsorption and Micellization
Source: Molecules. 2023 Mar 30;28(7):3115. doi: 10.3390/molecules28073115 (PMC10095903; doi:10.3390/molecules28073115)
Supplement: Supplementary file 1 [file molecules-28-03115-s001.zip › molecules-2288209-supplementary.pdf]

Supplementary Material

Thermodynamic parameters of berberine with Kolliphor mixtures adsorption and micellization

Magdalena Szaniawska, Katarzyna Szymczyk*, Anna Zdziennicka and Bronisław Jańczuk

Department of Interfacial Phenomena, Institute of Chemical Sciences, Faculty of Chemistry, Maria Curie-Skłodowska University in Lublin, Maria Curie-Skłodowska Sq. 3, 20-031 Lublin, Poland
*Correspondence: Katarzyna.szymczyk@mail.umcs.pl; Tel.: +48-81-537-55-38

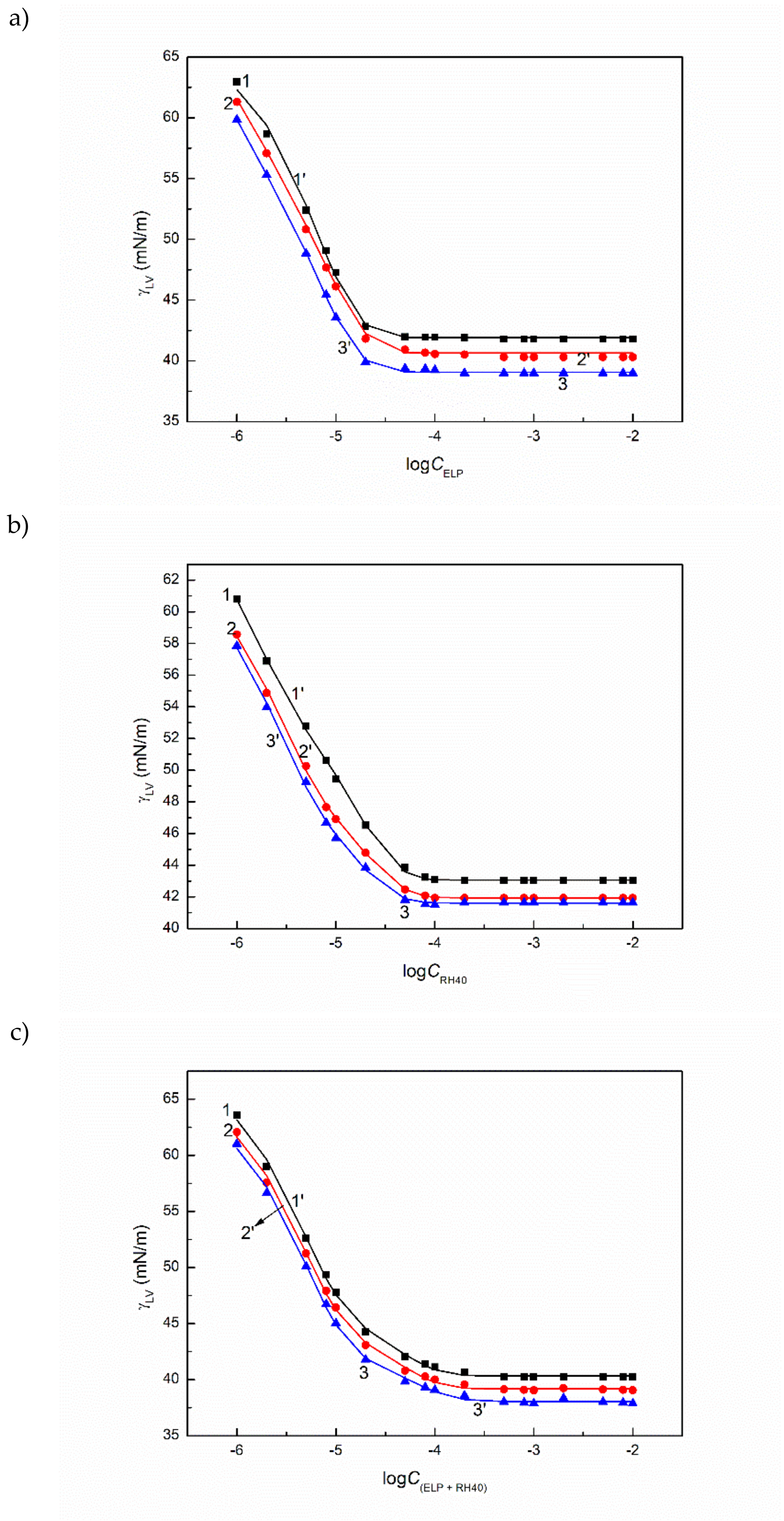

Figure S1. A plot of the surface tension (γ_{LV}) of M1 (a), M2 (b) and M3 (c) aqueous solutions vs. the logarithm of ELP ($\log C_{\mathrm{ELP}}$), RH40 ($\log C_{\mathrm{RH40}}$) and that of their concentration sum $\left(\log(C_{\mathrm{ELP}} + C_{\mathrm{RH40}})\right)$ at the constant temperature equal 293 K (points 1 and curve 1′), 303 K (points 2 and curve 2′) and 313 K (point 3 and curve 3′). Points 1 – 3 correspond to the measured values, curves 1′, 2′ and 3′ to the values calculated from Eq. (35).

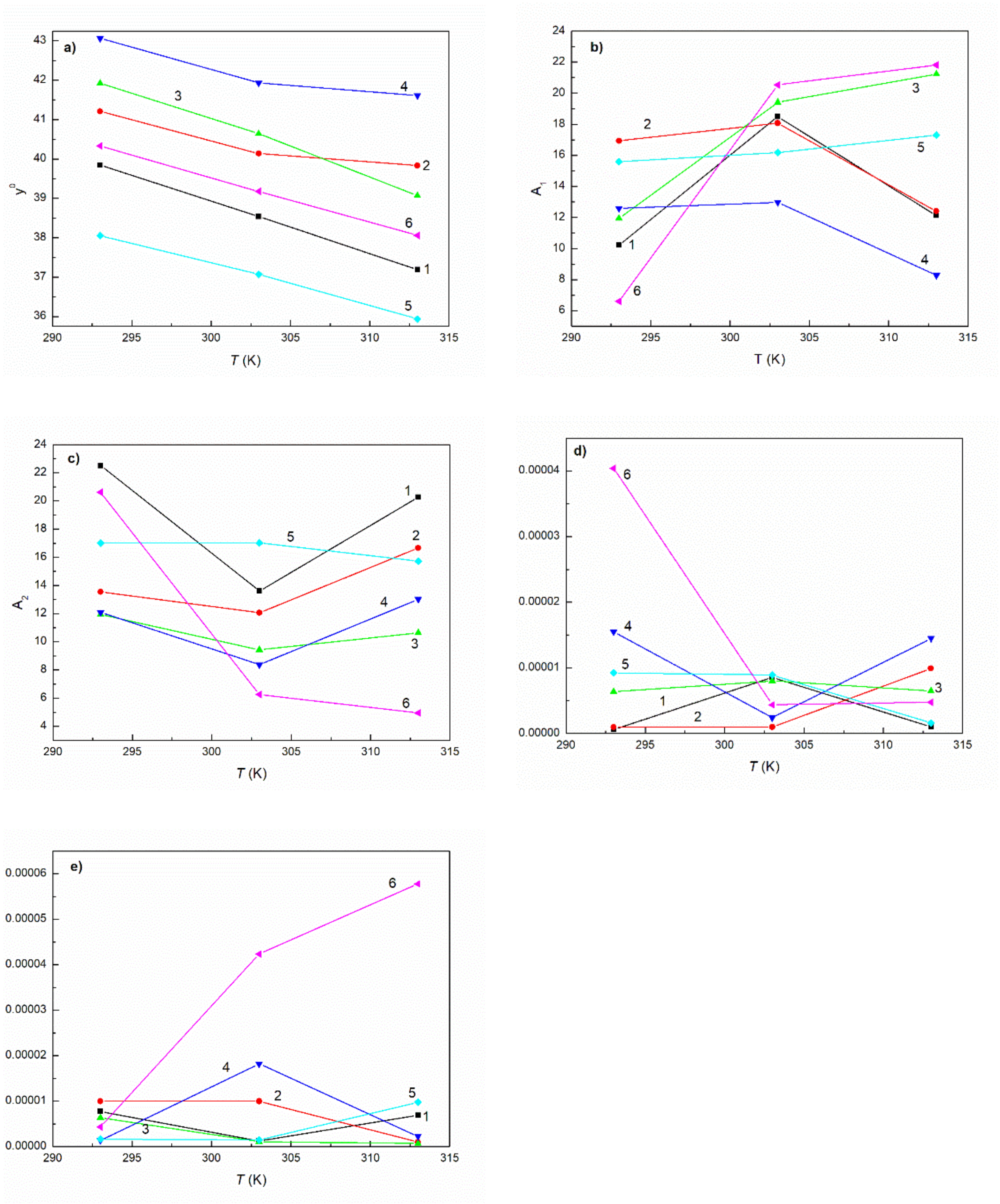

Figure S2. A plot of the constant y^0 (a), A_1 (b), A_2 (c), t_1 (d) and t_2 (e) in Eq. (35) vs. the temperature (T). Curves 1 – 6 correspond to the aqueous solutions of ELP, RH40, M1, M2, binary mixture of ELP and RH40 at the mole fraction of ELP in the bulk phase equal to 0.8 [22] and M3 mixtures, respectively.

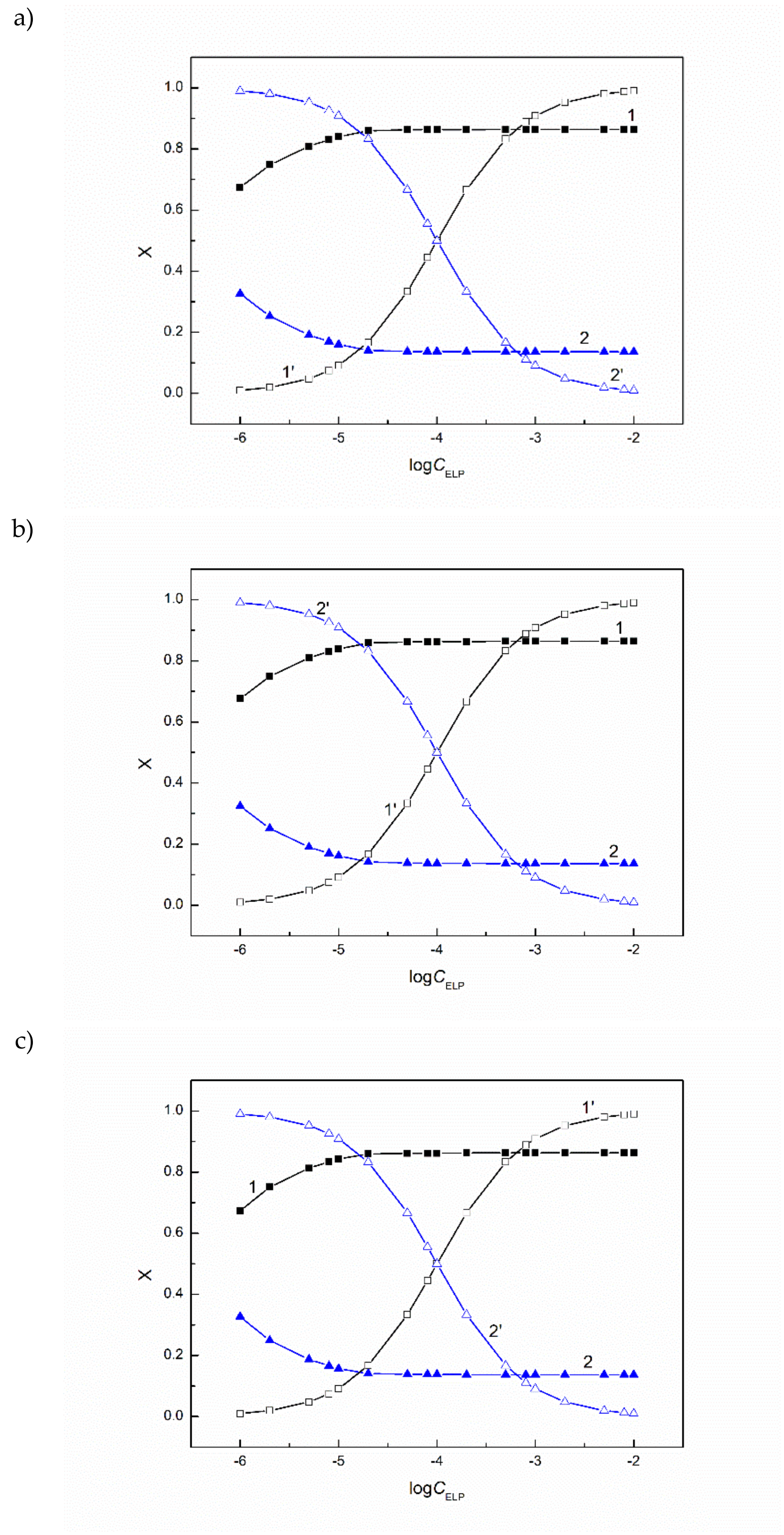

Figure S3. A plot of the mole fraction of ELP and Ber in M1 in the surface layer (curves 1 and 2) and in the bulk phase (curves 1′ and 2′) vs. the logarithm of ELP concentration ($\log C_{ELP}$) at the constant temperature equal 293 K (a), 303 K (b) and 313 K (c). Curves 1 and 1′ correspond to ELP, curves 2 and 2′ correspond to Ber.

a)

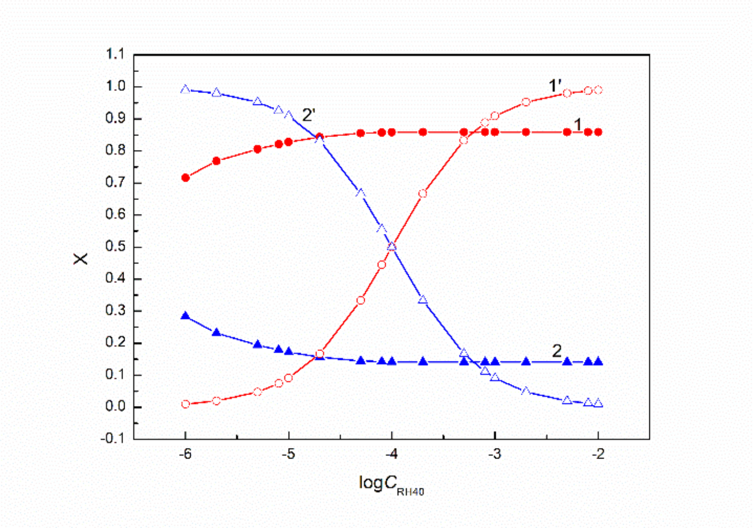

b)

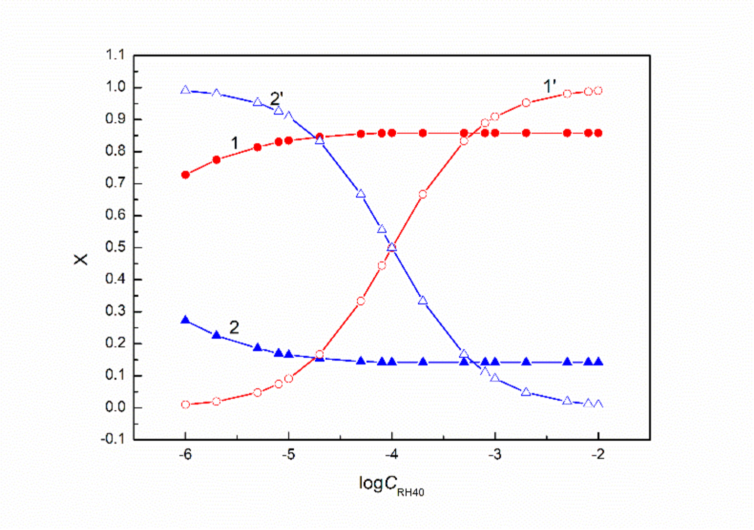

c)

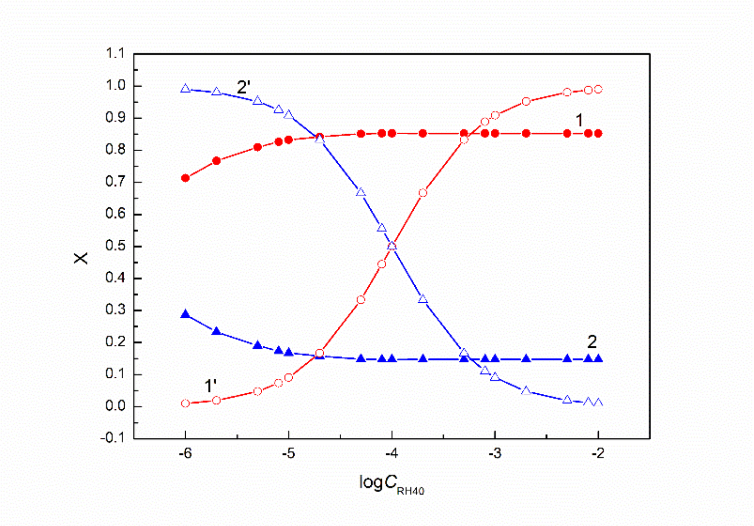

Figure S4. A plot of the mole fraction of RH40 and Ber in M2 in the surface layer (curves 1 and 2) and in the bulk phase (curves 1′ and 2′) vs. the logarithm of RH40 concentration ($\log C_{\mathrm{RH40}}$) at the constant temperature equal 293 K (a), 303 K (b) and 313 K (c). Curves 1 and 1′ correspond to RH40, curves 2 and 2′ correspond to Ber.

a)

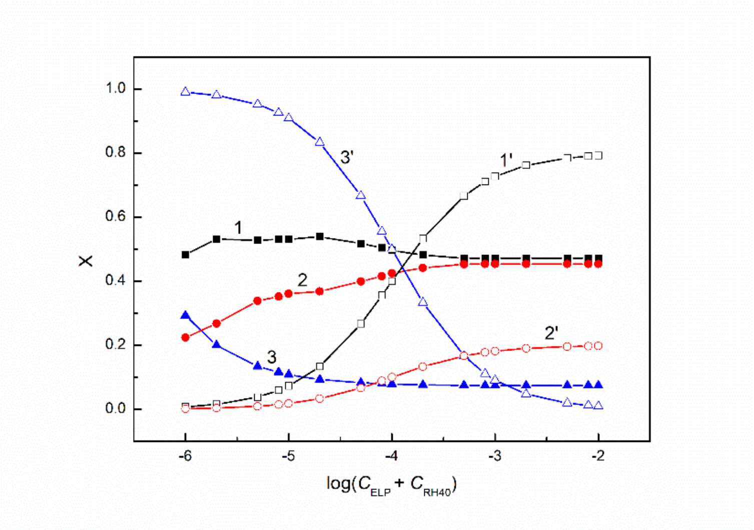

b)

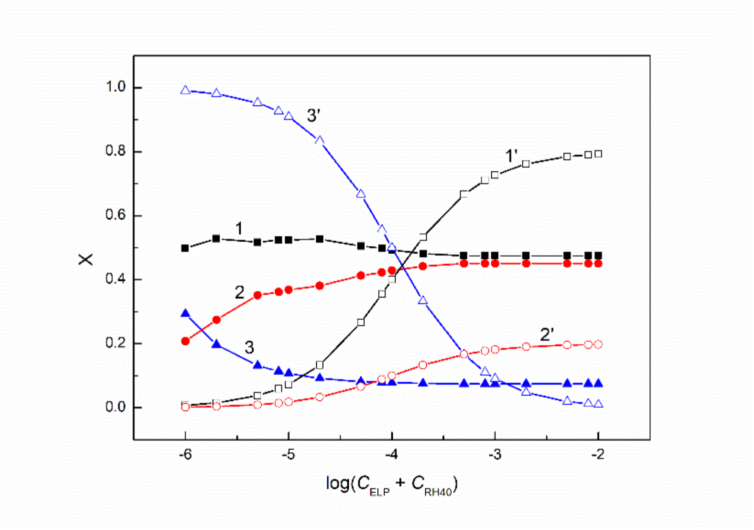

c)

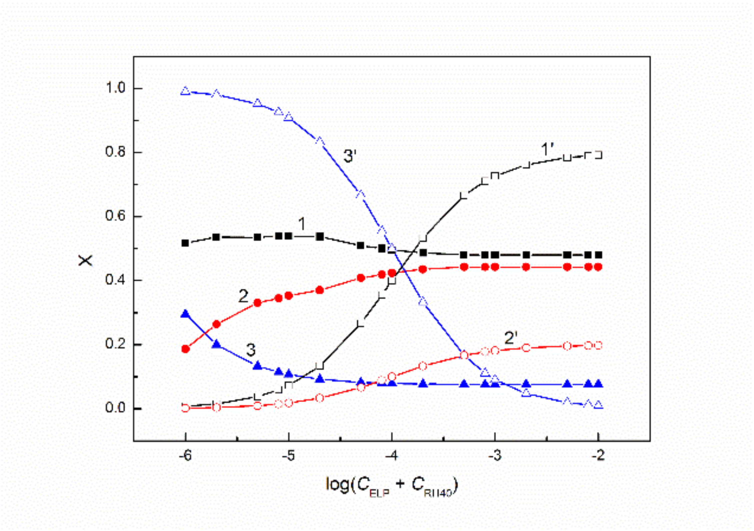

Figure S5. A plot of the mole fraction of ELP, RH40 and Ber in M3 in the surface layer (curves 1 -3) and in the bulk phase (curves 1 – 3′) vs. the logarithm of the sum of ELP and RH40 concentration log($C_{\mathrm{ELP}} + C_{\mathrm{RH40})}$ at the constant temperature equal 293 K (a), 303 K (b) and 313 K (c). Curves 1 and 1′ correspond to ELP, curves 2 and 2′ correspond to RH40 and curves 3 and 3′ correspond to Ber.

a)

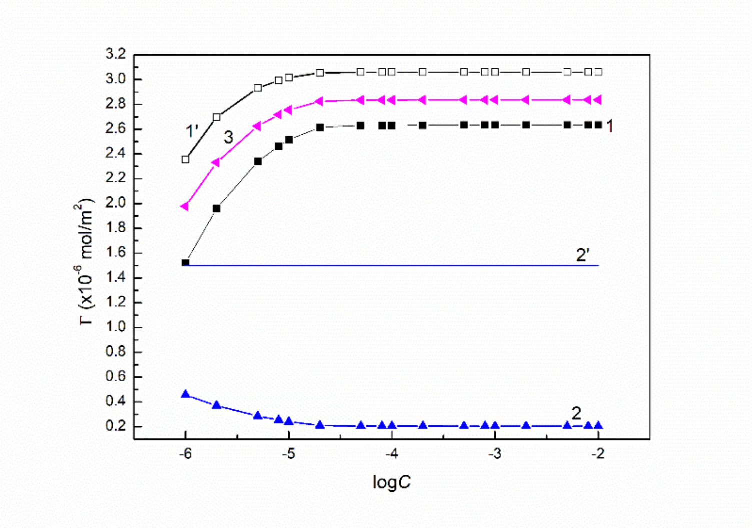

b)

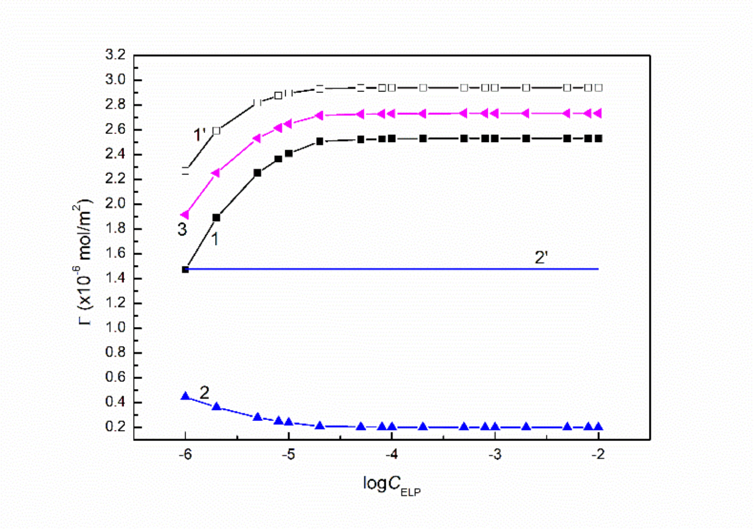

c)

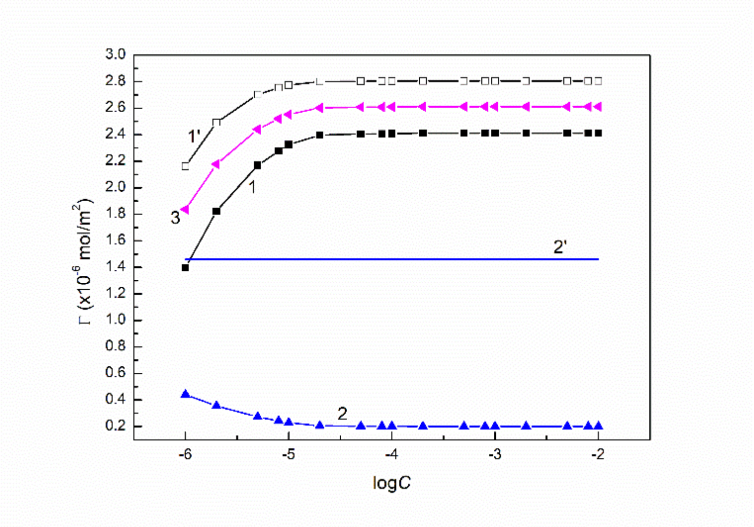

Figure S6. A plot of the surface concentration (Γ) calculated from Eq. (5) vs. the logarithm of ELP concentration ($\log C_{ELP}$) at the constant temperature equal 293 K (a), 303 K (b) and 313 K (c). Curves 1 and 2 correspond to the Γ values of ELP and Ber in M1 mixtures, curve 3 to their sum. Curves 1′ and 2′ correspond to the Γ values for ELP and Ber in their individual aqueous solutions.

a)

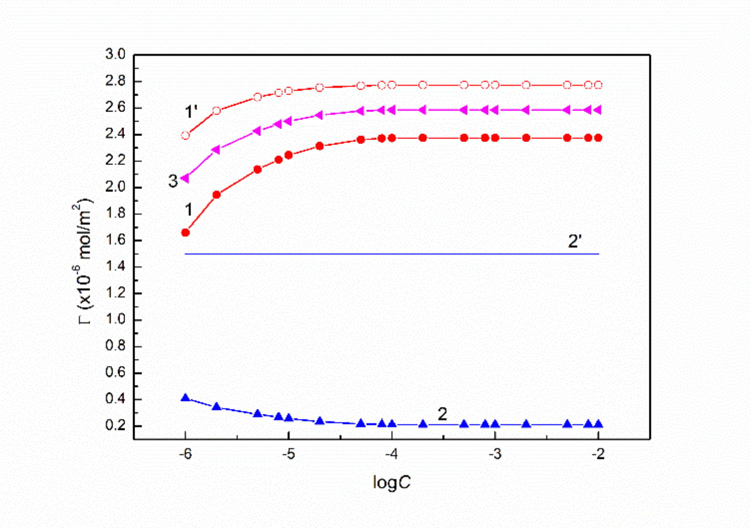

b)

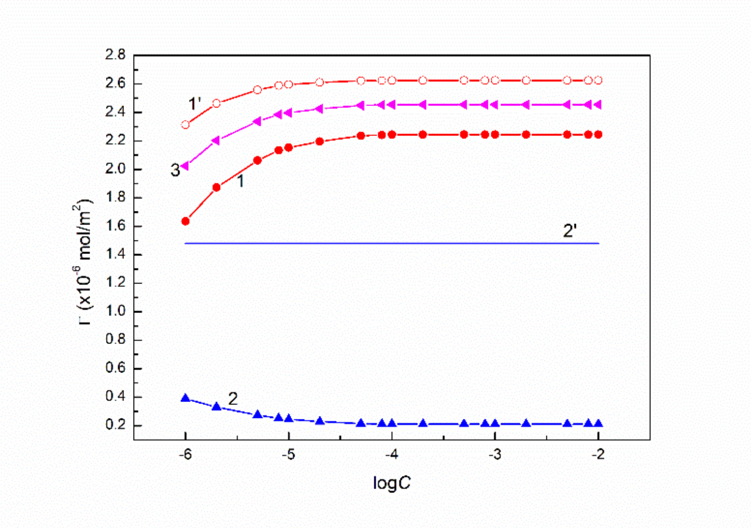

c)

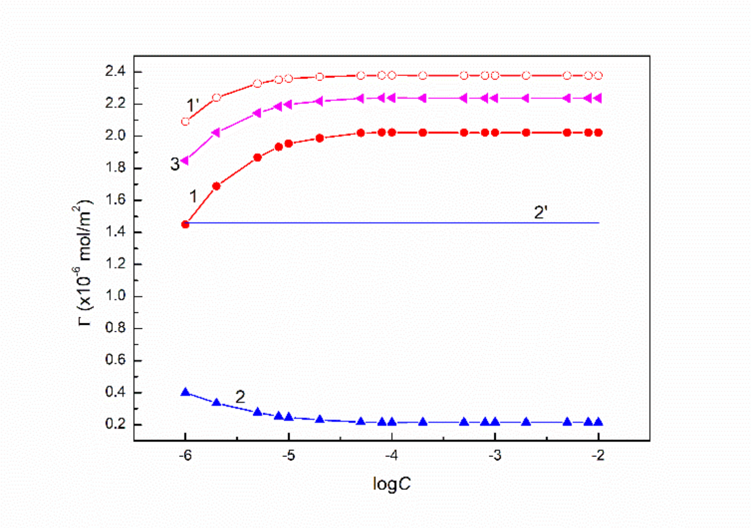

Figure S7. A plot of the surface concentration (Γ) calculated from Eq. (5) vs. the logarithm of RH40 concentration ($\log C_{\mathrm{RH40}}$) at the constant temperature equal 293 K (a), 303 K (b) and 313 K (c). Curves 1 and 2 correspond to the Γ values of RH40 and Ber in M2 mixtures, curve 3 to their sum. Curves 1′ and 2′ correspond to the Γ values for RH40 and Ber in their individual aqueous solutions.

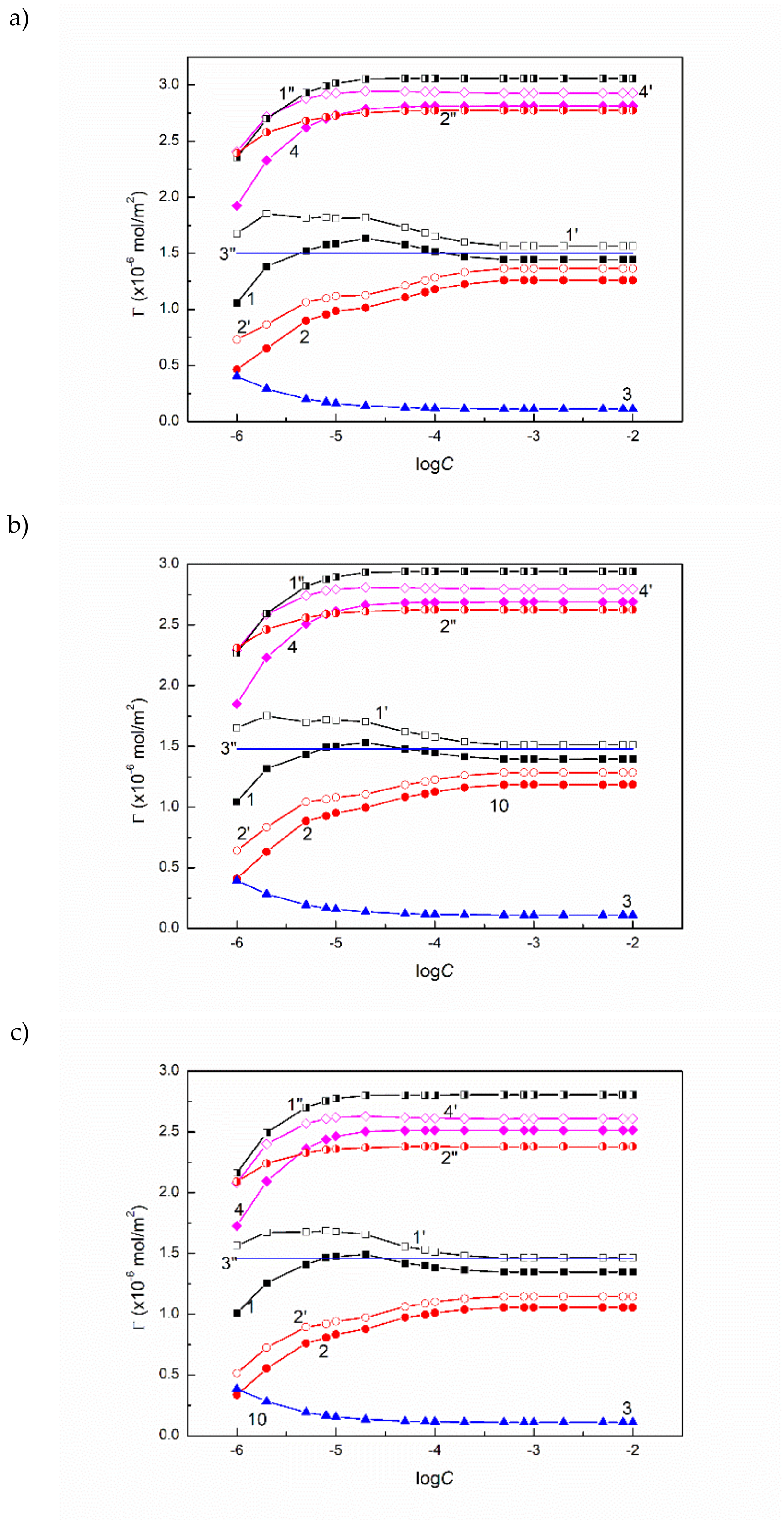

Figure S8. A plot of the surface concentration (Γ) calculated from Eq. (5) vs. the logarithm of concentration (log$C$) at the constant temperature equal 293 K (a), 303 K (b) and 313 K (c). Curves 1 – 4 correspond to the Γ values for ELP, RH40 and Ber in M3 mixtures and their sum. Curves 1′, 2′ and 5 correspond to the Γ values for ELP and RH40 in the 0.8ELP + RH40 mixtures and their sum. Curves 1′, 2′ and 3′ to the Γ values for ELP, RH40 and Ber in their individual aqueous solutions.

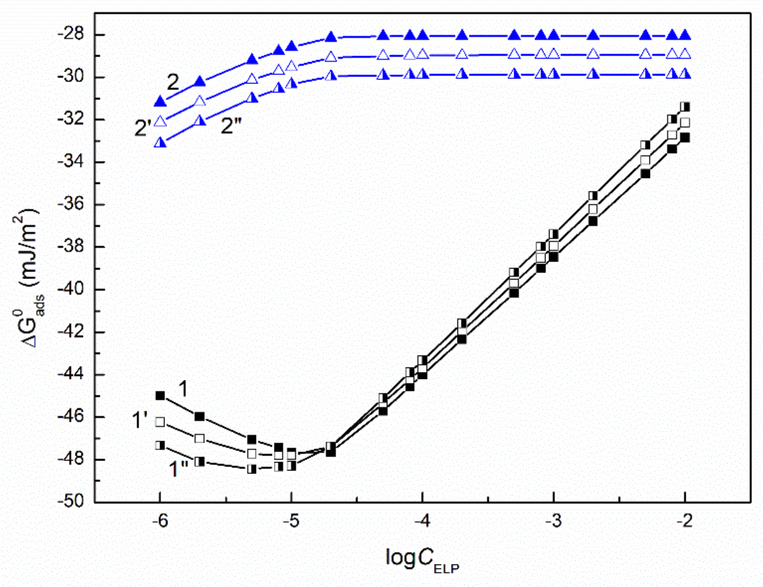

Figure S9. A plot of the Gibbs standard free energy of adsorption (ΔG^0_{ads}) calculated from Eq. (39) vs. the logarithm of ELP concentration ($\log C_{\mathrm{ELP}}$) at the constant temperature equal 293 K (curves 1 and 2), 303 K (curves 1′ and 2′) and 313 K (curves 1″ and 2″). Curves 1, 1′ and 1″ correspond to ELP, curves 2, 2′ and 2″ to Ber in M1 mixtures.

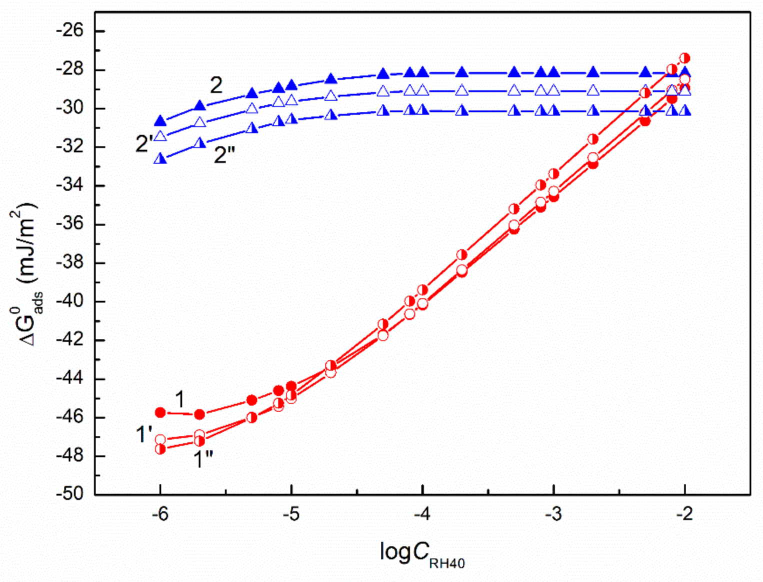

Figure S10. A plot of the Gibbs standard free energy of adsorption (ΔG^0_{ads}) calculated from Eq. (39) vs. the logarithm of RH40 concentration ($\log C_{\mathrm{RH40}}$) at the constant temperature equal 293 K (curves 1 and 2), 303 K (curves 1′ and 2′) and 313 K (curves 1″ and 2″). Curves 1, 1′ and 1″ correspond to RH40, curves 2, 2′ and 2″ to Ber in M2 mixtures.

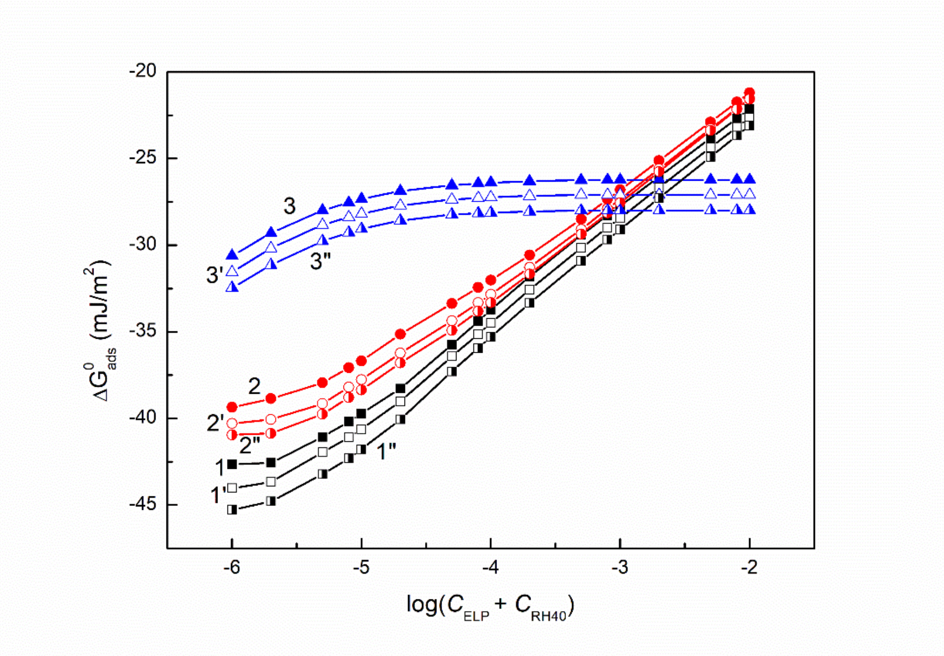

Figure S11. A plot of the Gibbs standard free energy of adsorption (ΔG^0_{ads}) calculated from Eq. (39) vs. the logarithm of the sum of ELP and RH40 concentration $\left(\log(C_{\mathrm{ELP}} + C_{\mathrm{RH40}})\right)$ at the constant temperature equal 293 K (curves 1 – 3), 303 K (curves 1′, 2′ and 3′) and 313 K (curves 1″, 2″ and 3″). Curves 1, 1′ and 1″ correspond to ELP, curves 2, 2′ and 2″ to RH40 and curves 3, 3′ and 3″ to Ber in M3 mixtures.
